# Supplementary material for: Shared biosynthetic architectures generate diverse β-amino polyketide residues in cyanobacterial peptides
Source: bioRxiv. 2026 Jun 4:2026.06.02.729631. Preprint. [Version 1] doi: 10.64898/2026.06.02.729631 (PMC13252402; doi:10.64898/2026.06.02.729631)
Supplement: Supplement 1 [file media-1.pdf]

Supporting Information for

Shared biosynthetic architectures generate diverse β-amino polyketide residues in cyanobacterial peptides

Aaditi Chopade,[a] Anmol Chaure,[b] Mary-Candler Schantz,[a] Runjie Xia,[a] David E. Berthold,[c] Forrest W. Lefler,[c] H. Dail Laughinghouse IV,[c] and Matthew J. Bertin[a,*]

[a]Department of Chemistry, Case Western Reserve University, Cleveland, OH 44106, United States

[b]Faculty of Environmental Sciences, Czech University of Life Sciences, Prague, Czech Republic

[c]Fort Lauderdale Research and Education Center, University of Florida – Institute of Food and Agricultural Sciences, Davie, FL 33314, United States

Corresponding Author Email: mxb1224@case.edu

This PDF file includes:

Table S1-S6
Figures S1-S32

Table S1. NMR data for floridanemamide A (**1**) (500 MHz for ^{1}H and 125 MHz for ^{13}C, DMSO-d_6)

Unit	No	δ_C, multi.	δ_H [mult., J (Hz)]	HMBC	TOCSY
Pro-1	1	171.4, C			
	2	55.7, CH	4.27, ovlp	1	2, 3a, 3b, 5b
	3a	27.8, CH_2	1.98, m		2, 3b, 5b
	3b		1.72, m		3a, 3b, 5b
	4	23.9, CH_2	1.85, ovlp		2
	5a	46.7, CH_2	3.50, m		2, 3a, 3b
	5b		3.33, m		3b
D-*O*-me-Htyr	6	169.6, C			
	7	50.4, CH	4.45, m	6	NH, 8, 9a, 9b
	8	33.0, CH_2	1.88, m	9	NH, 7, 9a, 9b
	9a	30.2, CH_2	2.54, m	8	NH, 7, 8, 9b
	9b		2.44, m		NH, 7, 8
	10	133.4, C			
	11	129.2, CH	7.10, d, 8.5	10, 13	12
	12	113.7, CH	6.82, d, 8.5	10, 13	11
	13	157.3, C			
	14	113.7, CH	6.82, d, 8.5	10, 13	15
	15	129.2, CH	7.10, d, 8.5	10, 13	14
	16	55.0, CH_3	3.70, s	13	
	NH		7.93, m		7, 8, 9a, 9b
Thr	17	169.9, C			
	18	58.9, CH	4.35, ovlp	17	NH, 20
	19	66.8, CH	4.22, m		20
	20	20.2, CH_3	1.07, d, 6.3	19	NH, 18, 19
	NH		7.82, m		18, 19, 20
Dhb	21	163.9, C		23	
	22	131.5, C		23	
	23	120.6, CH	5.70, m	21	24
	24	13.1, CH_3	1.81, d, 7.3	23	NH
	NH		9.35, m		23, 24
Ser-1	25	170.5, C			
	26	56.6, CH	4.16, m	25	NH, 27a, 27b
	27a	61.8, CH_2	3.69, m	26	NH
	27b		3.55, m		NH
	NH		8.14, m		26, 27a, 27b
D-*allo*-Ile	28	171.0, C			
	29	58.5, CH	4.47, m	28	NH, 30
	30	35.3, CH	1.85, ovlp	33	NH, 32, 33
	31	25.3, CH_2	1.00, m	33	32, 33
	32	11.6, CH_3	0.74, t, 7.3	31	NH, 30, 31

	33	14.5, CH_3	0.69, d, 6.4	31	
	NH		8.60, m		29, 30, 31, 32, 33
Phe	34	172.0			
	35	54.7, CH	4.50, m	34	NH, 36a, 36b
	36a	36.5, CH_2	3.01, m	35	NH, 35, 36a
	36b		2.97, m		
	37	138.4, C			
	38	129.3, CH	7.23, m	37	
	39	128.2, CH	7.27, m		
	40	126.7, CH	7.24, m	39, 41	
	41	128.2, CH	7.27, m		
	42	129.3, CH	7.23, m	37, 41	
	NH		8.11, m		35, 36
Ser-2	43	170.7, C			
	44	55.7, CH	4.32, ovlp	43	NH, 45 a
	45a	61.3, CH_2	3.72, m		NH, 44
	45b		3.57, m		NH, 44
	NH		8.12, m		44, 45b
Gly	46	170.3, C			
	47	41.7, CH_2	3.78, m	46	NH
	NH		8.20, m		47
D-Gln	48	172.0, C			
	49	52.4, CH	4.24, ovlp	48	NH, 50, 51
	50	28.7, CH_2	1.92, m	51	NH, 49, 51
	51	31.5, CH_2	2.09, m	52	NH, 49,50
	52	174.9, C			
	NH_2	n.o.[a]			
	NH		7.95, m		49, 50, 51
Athmu	53	171.5, C			
	54	72.0, CH	3.94, m	53	NH, 56a
	55	48.7, CH	4.09, m		NH, 54, 56 a, 56b, 57, 59
	NH		7.60, m		54, 55, 56a, 56b, 57, 58
	56a	37.9, CH_2	1.77, ovlp		NH, 55, 58, 60
	56b		1.43, m		NH, 55, 58, 60, 61, 63
	57	69.9, CH	4.97, m		NH, 55, 56a, 56b, 58, 59
	58	38.0, CH_2	1.47, m		NH, 54, 55, 56a, 56b, 60, 61, 63, 64

	59	71.2, CH	3.20, m		57, 58, 61, 62, 63, 64
	60	71.5, CH	3.29, m	59	57, 58, 61, 62, 63, 64
	61	41.7, CH_2	1.19, m	62	60, 62, 63, 64
	62	23.5, CH	1.75, ovlp	61	NH, 55, 58, 60, 61, 63
	63	23.9, CH_3	0.86, ovlp	62	58, 60, 61, 62, 64
	64	21.7, CH_3	0.81, d, 6.6	62	63
Pro-2	65	172.2		57, 66	
	66	58.4, CH	4.46 m	67	67a
	67a	28.6, CH_2	1.93, m		66
	67b		1.79, ovlp		67a
	68	21.7, CH_2	1.75, ovlp		67a
	69a	46.0, CH_2	3.46, m		67a, 68
	69b		3.29, m		67b, 68
Butyric acid	70	171.7, C			
	71	35.7, CH_2	2.24, m	70, 72	72, 73
	72	18.1, CH_2	1.48, m	70, 73	71, 73
	73	13.6, CH_3	0.87, ovlp		71, 72

[a]not observed

Table S2. NMR data for floridanemamide B (**2**) (500 MHz for ^{1}H and 125 MHz for ^{13}C, DMSO-d_6)

Unit	No	δ_c, multi.	δ_H [mult., J (Hz)]
Pro-1	1	167.0, C	
	2	60.2, CH	4.27, m
	3	28.0, CH_2	1.93, m
	4	24.8, CH_2	1.86, m
	5a	46.7, CH_2	3.48, m
	5b		3.34, m
D-*O*-me-Htyr	6	166.9, C	
	7	51.5, CH	4.32, m
	8a	33.4, CH_2	1.87, m
	8b		1.70, m
	9a	30.7, CH_2	2.55, m
	9b		2.41, m
	10	133.8, C	
	11	129.5, CH	7.10, d, 8.3
	12	113.7, CH	6.82, d, 8.3
	13	157.5, C	
	14	113.7, CH	6.82, d, 8.3
	15	129.5, CH	7.10, d, 8.3
	16	55.3, CH_3	3.70, s
	NH		7.97, m
Thr	17	n.o.[a]	
	18	59.8, CH	4.26, m
	19	66.9, CH	4.14, m
	20	20.1, CH_3	1.08, d, 6.3
	NH		7.48, m
Dhb-1	21	164.3	
	22	141.2	
	23	124.4, CH	5.64, q, 7.2
	24	13.2, CH_3	1.83, s
	NH		9.28, m
Ser-1	25	172.4, C	
	26	56.5, CH	4.22, m
	27a	61.4, CH_2	3.77, m
	27b		3.69, m
	NH		8.00, m
Dhb-2	28	164.9, C	
	29	140.0, C	
	30	129.7, CH	5.76, m
	31	13.2, CH_3	1.83, s
	NH		9.02, m
Phe	32	170.6, C	

	33	56.48, CH	4.35, m
	34a	36.7, CH_2	3.04, m
	34b		1.61, m
	35	137.5, C	
	36	129.0, CH	7.29, ovlp
	37	129.0, CH	7.29, ovlp
	38	126.7, CH	7.23, m
	39	129.0, CH	7.29, ovlp
	40	129.0, CH	7.29, ovlp
	NH		7.99, m
Pro-2	41	172.8, C	
	42	58.8, CH	4.41, m
	43	28.2, CH_2	1.88, m
	44	21.8, CH_2	1.66, m
	45	46.0, CH_2	3.47, m
Gly	46	168.5, C	
	47	41.2, CH_2	3.87, m
	NH		7.95, m
D-Gln	48	170.2, C	
	49	52.8, CH	4.20, m
	50	28.4, CH_2	1.92, m
	51	31.5, CH_2	2.11, m
	52	174.5, C	
	NH_2		n.o.
	NH		7.67, m
Athmu	53	171.0, C	
	54	72.0, CH	3.86, m
	55	48.6, CH	4.00, m
	NH		7.56, m
	56	36.7, CH_2	1.76, m
	57	69.6, CH	5.03, m
	58	37.7, CH_2	1.41, m
	59	70.9, CH	3.14, m
	60	72.0, CH	3.27, m
	61	41.9, CH_2	1.18, m
	62	23.9, CH	1.71, m
	63	21.6, CH_3	0.83, ovlp
	64	23.4, CH_3	0.88, ovlp
N-Me-Leu	65	170.5, C	
	66	53.7, CH	5.08, m
	67	36.3, CH_2	1.62, m
	68	24.3, CH	1.38, m
	69	21.3, CH_3	0.87, ovlp

	70	23.2, CH_3	0.89, ovlp
	71	32.0, CH_3	2.82, s
Ac	72	170.7. C	
	73	21.6, CH_3	2.00, s

[a]not observed

Table S3. NMR data for floridanemamide C (**3**) (500 MHz for ^{1}H and 125 MHz for ^{13}C, DMSO-d_6)

Unit	No	δ_C, multi.	δ_H [mult., J (Hz)]	HMBC	TOCSY
Pro-1	1				
	2	58.5, CH	4.25, m		3, 4, 5a
	3	28.4, CH_2	2.06, m		5a
	4	24.3, CH_2	1.87, ovlp		2, 5a
	5a	46.9, CH_2	3.47, m		2, 3, 4
	5b		3.34, m		
D-*O*-me-Htyr	6				
	7	51.2, CH	4.45, m		8, 9a, NH
	8	33.9, CH_2	1.88, m		7, 9a, NH
	9a	30.9, CH_2	2.56, m	8, 10	7, 8, NH
	9b		2.50, m		
	10	134.7, C			
	11	129.3, CH	7.10, d, 8.3	13	12
	12	113.6, CH	6.81, d, 8.3	10	11
	13	157.9, C			
	14	113.6, CH	6.81, d, 8.3	10	15
	15	129.3, CH	7.10, d, 8.3	13	14
	16	55.3, CH_3	3.71, s	13	
	NH		8.01, ovlp		7
Thr	17				
	18	57.6, CH	4.39, m		19, 20, NH
	19	67.2, CH	4.04, m		20, NH
	20	19.9, CH_3	1.04, d, 6.0	19	18, 19
	NH		8.17, m		18, 19
Leu	21				
	22	55.3, CH	4.46, m		NH, 23, 24, 25, 26
	23	38.3 CH_2	1.78, m		NH, 22, 24, 25, 26
	24	24.1, CH	1.56, m		22, 23, 25, 26
	25	19.4, CH_3	0.85, ovlp	24	22, 23, 24
	26	19.2, CH_3	0.79, ovlp	24	22, 23, 24
	NH		8.00, ovlp		22, 23
Ser-1	27				
	28	56.3, CH	4.28, m		29a, 29b, NH
	29a	62.4, CH_2	3.65, m		28, NH
	29b		3.58, m		28, NH
	NH		8.01, ovlp		28, 29a, 29b
D-*allo*-Ile	30				
	31	58.6, CH	4.47, m		33, 34, 35, NH
	32	36.3, CH	1.97, m		33, 34, 25

	33	25.9, CH_2	1.12, m		32, 34, 35
	34	11.6, CH_3	0.82, t, 7.0	33	32, 33
	35	14.7, CH_3	0.79, d, 7.2	33	32, 33
	NH		8.30, m		31
Val-1	36	170.2, C			
	37	59.5, CH	4.11, m		NH, 38, 39, 40
	38	30.5, CH	1.98, ovlp		NH, 37, 39, 40
	39	19.0, CH_3	0.88, d, 6.7	38	37, 38
	40	18.8, CH_3	0.92, d, 6.7	38	37, 38
	NH		8.23, m		37, 38, 39, 40
Gln	41				
	42	52.3, CH	4.24, m		NH, 43, 44
	43	28.5, CH_2	1.88, ovlp		NH, 42, 44
	44	32.0, CH_2	2.09, m	43, 44	NH, 42, 43
	45	174.9, C			
	NH		7.95, s		42
	NH_2		8.01, br	45	
Gly	46	168.5, C			
	47	41.7, CH_2	3.72, m		NH
	NH		8.22, m		47
D-Val-2	48	170.2, C			
	49	58.8, CH	3.98, m		NH, 50, 51, 52
	50	36.2, CH	1.98, ovlp		NH, 49, 51, 52
	51	19.0, CH_3	0.87, ovlp		49, 50
	52	19.0, CH_3	0.88, ovlp	50	49, 50
	NH		7.79, m	50	49, 50, 51, 52
Athmu	53	171.2, C			
	54	73.2, CH	3.97, m		NH, 56, 59, 60
	55	48.8, CH	4.05, m		NH, 56, 57, 59, 60
	NH		7.43, s		NH, 55, 56
	56	37.9, CH_2	1.78, ovlp		NH, 57, 59, 60, 61
	57	69.6, CH	5.03, m		NH, 55, 56, 58
	58	38.0, CH_2	1.41, m		57, 59, 62
	59	70.5, CH	3.26, ovlp		58, 61, 62, 63, 64
	60	71.6, CH	3.29, ovlp		61, 62, 63, 64
	61	41.9, CH_2	1.20, m	62	60, 62, 63, 64
	62	24.0, CH	1.76, ovlp		60, 61, 63, 64
	63	21.6, CH_3	0.82, ovlp	62	62
	64	24.0, CH_3	0.86, ovlp	62	62
Pro-2	65	170.5, C			
	66	53.7, CH	4.47, m		67, 68, 69a

	67	28.0, CH_2	1.85, m		66, 69, 69b
	68	22.7 CH_2	1.75, m		66, 67, 69a, 69b
	69a	46.9 CH_2	3.49, ovlp		66, 67, 68
	69b		3.31, ovlp		68
Butyric acid	70	172.8, C			
	71	35.5, CH_2	2.22, m		72, 73
	72	17.7, CH_2	1.50, m	71, 73	71, 73
	73	13.9, CH_3	0.86, m	72	71, 72

Table S4. Predicted Protein Annotations for *fma*, *fmb*, and *fmc* Biosynthetic Pathways

ORF	Module	Size (nt)	Proposed Domain Organization/ Function (AntiSMASH)	Predicted Substrate	Similar Sequence	Identity (%)	Coverage (%)	E-value	Accession number
**Floridanemamide A (BLCC F50)**									
***fma*A**	Loading	13,587	A, KR, ACP	α-ketoisocaproic acid	*Microseira wollei]*	71.61	99	0	WP_226584072.1
	1		KS, AT, KR, ACP	malonyl CoA					
	2		KS, AT, KR, ACP	malonyl CoA					
***fma*B**	1	10,890	KS, AT, AmT, MO	malonyl CoA	*Desmonostoc muscorum* CCALA 125	72.0	100	0	UBH04462.1
	2		C, A, E, PCP	D-Gln					
***fma*C**	1	24,054	C, A, PCP	Gly	*Desmonostoc muscorum* CCALA 125	60.3	100	0	UBH04463.1
	2		C, A, PCP	L-Ser					
	3		C, A, PCP	L-Phe					
	4		C, A, E, PCP	D-*allo*-Ile					
	5		C, A, PCP	L-Ser					
	6		C, A, PCP	L-Thr					
	7		C, A, PCP	L-Thr					
***fma*D**	1	9,900	C, A, E, oMT, PCP	D-hTyr	*Desmonostoc muscorum* LEGE 12446	67.02	89	0	WP_255264360.1
	2		C, A, PCP	L-Pro					
***fma*E**	1	4173	C, A, PCP, TE	L-Pro	*Nostoc* sp.	59.97	99	0	WP_335055746.1
**Floridanemamide B (BLCC F306)**									
***fmb*A**	Loading	13,485	A, KR, ACP	α-ketoisocaproic acid	*Microseira wollei]*	71.49	99	0	WP_226584072.1
	1		KS, AT, KR, ACP	malonyl CoA					
	2		KS, AT, KR, ACP	malonyl CoA					
***fmb*Bp**	1	10,857	KS, AT, AmT, MO	malonyl CoA	*Desmonostoc muscorum* CCALA 125	72.60	100	0	UBH04462.1
	2		C, A, E, PCP	D-Gln					
***fmb*C**	1	22,584	C, A, PCP	Gly	*Desmonostoc muscorum* CCALA 125	63.47	100	0	UBH04462.1
	2		C, A, PCP	L-Pro					
	3		C, A, PCP	L-Phe					
	4		C, A, E, PCP	L-Thr					
	5		C, A, PCP	L-Ser					
	6		C, A, PCP	L-Thr					
	7		C, A, PCP	L-Thr					
***fmb*D**	1	9,687	C, A, E, oMT, PCP	D-hTyr	*Desmonostoc muscorum* LEGE 12446	68.43	89	0	WP_255264360.1
	2		C, C, A, PCP	L-Pro					
***fmb*E**	1	4,743	C, A, nMT, PCP, TE	L-Leu	*Nostoc* sp. UHCC 0251	62.63	83	0	WP_323199853.1
**Floridanemamide C (BLCC F167)**									
***fmc*A**	Loading	13,557	A, KR, ACP	α-ketoisocaproic acid	*Microseira wollei*	71.06	99	0	WP_226584072.1
	1		KS, AT, KR, ACP	malonyl CoA					
	2		KS, AT, KR, ACP	malonyl CoA					
***fmc*B**	1	11,061	KS, AT, AmT, MO	malonyl CoA	*Desmonostoc muscorum* CCALA 125	69.05	100	0	UBH04462.1
	2		C, A, E, PCP	D-Val					
***fmc*C**	1	25,200	C, A, PCP	Gly	*Desmonostoc muscorum* LEGE 12446	66.74	100	0	WP_255264359.1
	2		C, A, PCP	L-Gln					
	3		C, A, PCP	L-Val					
	4		C, A, E, PCP	D-*allo*-Ile					
	5		C, A, PCP	L-Ser					
	6		C, A, PCP	D-Leu					
	7		C, A, PCP	L-Thr					
***fmc*D**	1	9,795	C, A, E, oMT, PCP	D-hTyr	*Desmonostoc muscorum* LEGE 12446	67.35	89	0	WP_255264360.1
	2		C, A, PCP	L-Pro					
***fmc*E**	1	4,188	C, A, PCP, TE	L-Pro	Unclassified *Microcystis* (*IN: CYANOBACTERIA)*	64.77	70	0	WP_287686773.1

Table S5. Residue composition of cyanopeptides with beta amino acids

**Compound Name**	**β-aa**	**1**	**2**	**3**	**4**	**5**	**6**	**7**	**8**	**9**	**10**	**Post-assembly line tailoring**
Tychonamide	Atpoa	D-Gln	Gly	Pro	Pro	D-*allo*-Ile	Ser	Dhb	Thr	D-O-Me-Htyr	Pro	N-Ac-N-Me-Leu
Floridanemamide A	Athmu	D-Gln	Gly	Ser	Phe	D-*allo*-Ile	Ser	Dhb	Thr	D-O-Me-Htyr	Pro	N-Butyl-Pro
Floridanemamide B	Athmu	D-Gln	Gly	Pro	Phe	Dhb	Ser	Dhb	Thr	D-O-Me-Htyr	Pro	N-Ac-N-Me-Leu
Pahayokolide A	Athmu	D-Gln	Gly	Pro	Phe	Dhb	Ser	Dhb	Thr	D-HPhe	Pro	N-Ac-N-Me-Leu
Portoamide A/Lyngbyazothrin C	Athmu	D-Gln	Gly	Pro	Pro	D-*allo*-Ile	Ser	Dhb	Thr	D-O-Me-Htyr	Pro	N-Ac-N-Me-Tyr
Floridanemamide C	Athmu	D-Val	Gly	Gln	Val	D-*allo*-Ile	Ser	D-Leu	Thr	D-O-Me-Htyr	Pro	N-Butyl-Pro
Schizotrin A	Athmu	D-Gln	Gly	Pro	Phe	D-Val	Ser	Dhb	Ser	O-Me-Htyr	Pro	N-Butyl-N-Me-D-Ala
Scytonemin A	Ahda	D-Ser	Gly	HyMePro	HyMePro	D-Leu	Hser	D-Phe	Gly	D-HyLeu	MePro	N-Ac-Ala
Muscotoxin A	Ahdoa	D-Gln	Gly	Pro	Phe	D-*allo*-Ile	Ser	Dhb	Ser	D-*allo*-Ile	Pro	

Table S6. Genomic information for analyzed cyanobacterial strains of *Floridanema*

Strain	*F. flaviceps* BLCC-F50	*F. evergladense* BLCC-F167	*F. aerugineum* BLCC-F306
Completion (%)	99.94	100	100
Contamination (%)	0.1	0.05	0.06
Coverage Illumina (x)	29.48	19.6	124.1
Coverage ONT (x)	88.8	60.1	84.8
Contigs	3	1	12
Genome Size (bp)	6750723	6463832	6967955
GC %	40.7	40.1	40.5
tRNA	73	75	76
rRNA	9	9	9
CDS	6196	5981	6234
SRA	SRS22629764	SRS22630153	SRS29217320
BioSample	SAMN43549591	SAMN43549593	SAMN60208900

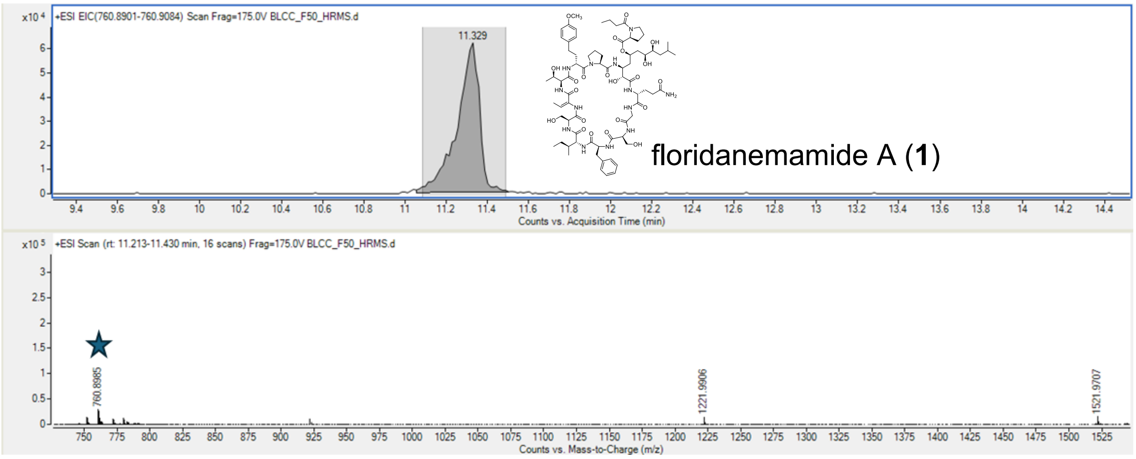

Figure S1. HRESIMS measurement of floridanemamide A (**1**) *m/z* 760.8985 $[M+2H]^{2+}$.

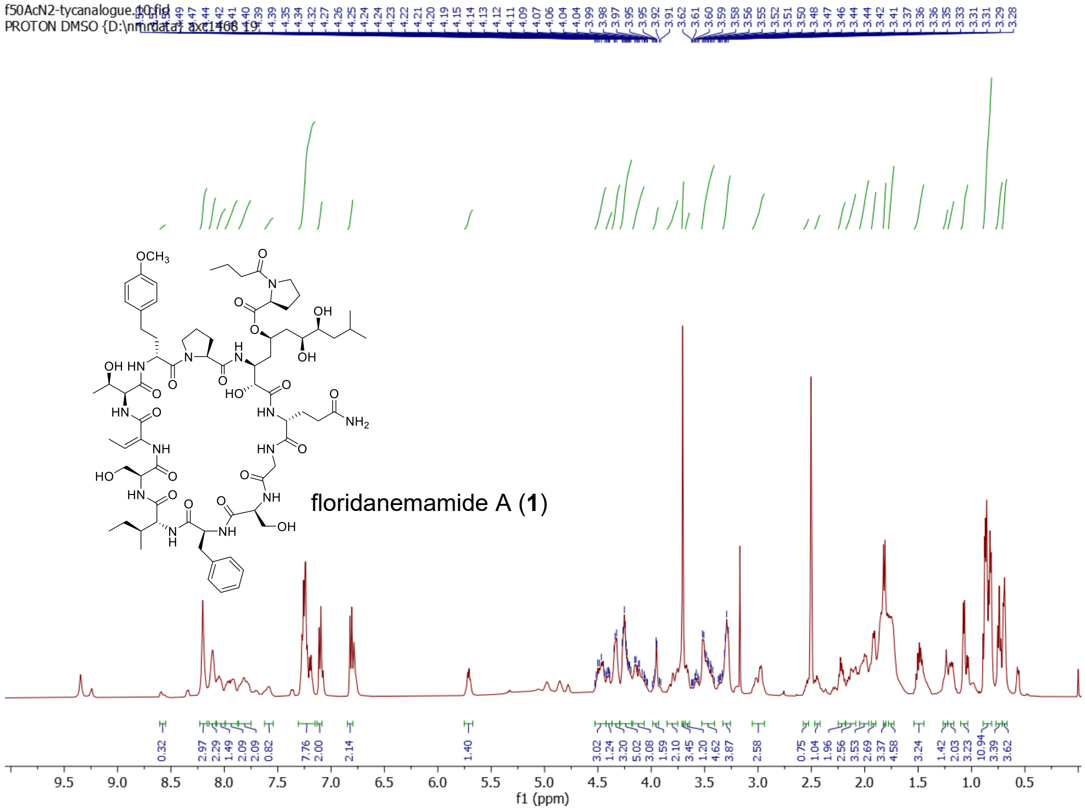

Figure S2. ^{1}H NMR of floridanemamide A (**1**) (500 MHz, DMSO-d_6).

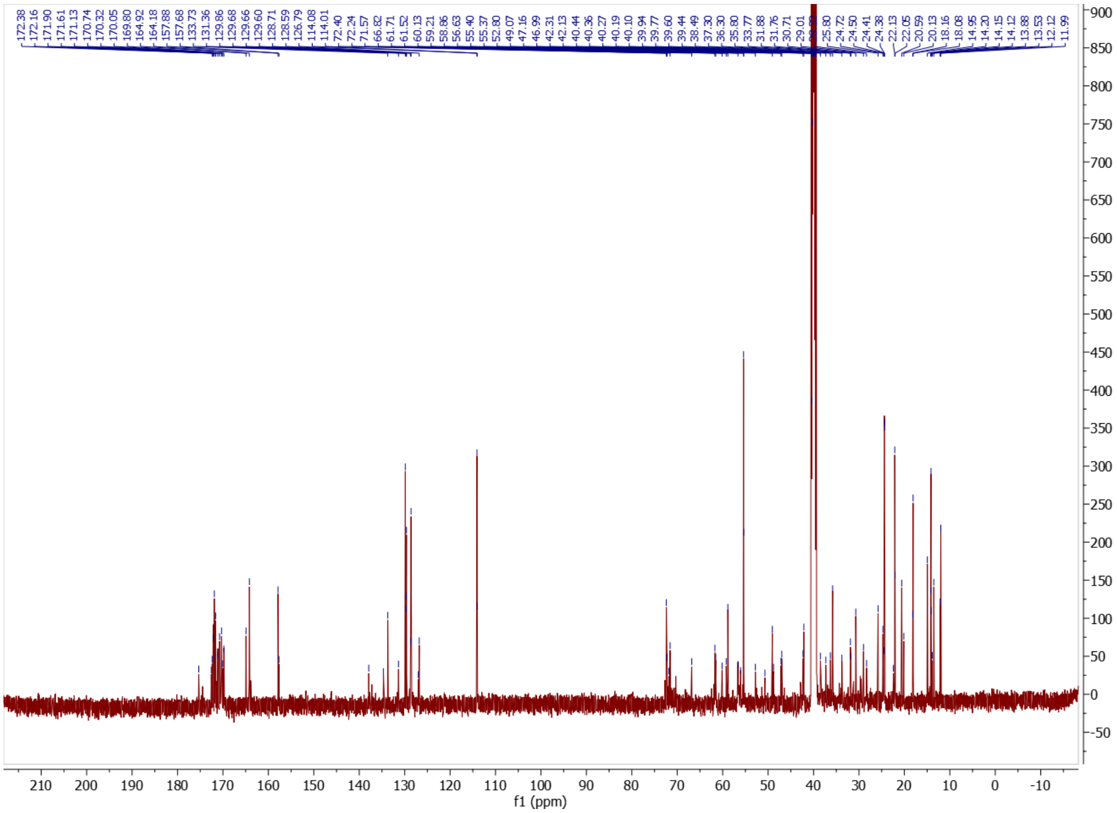

Figure S3. ^{13}C NMR of floridanemamide A (125 MHz, DMSO-d_6).

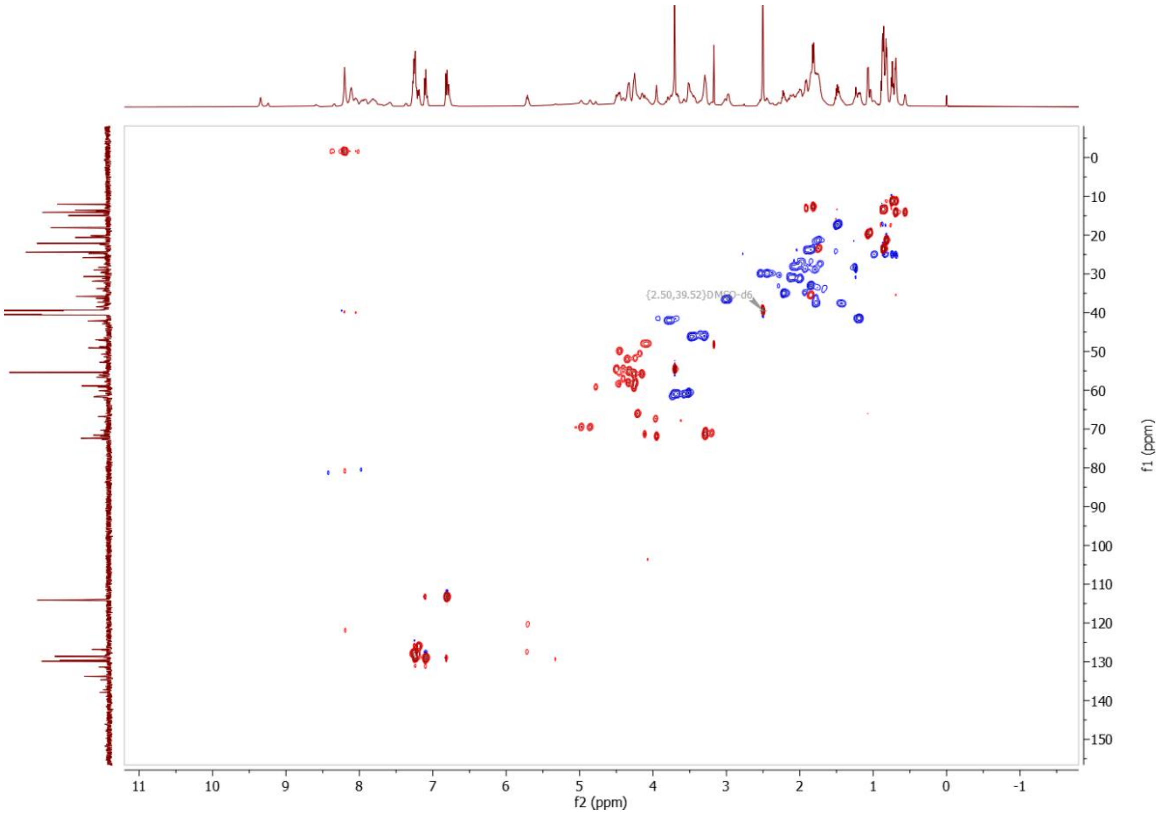

Figure S4. Multiplicity-edited HSQC of floridanemamide A.

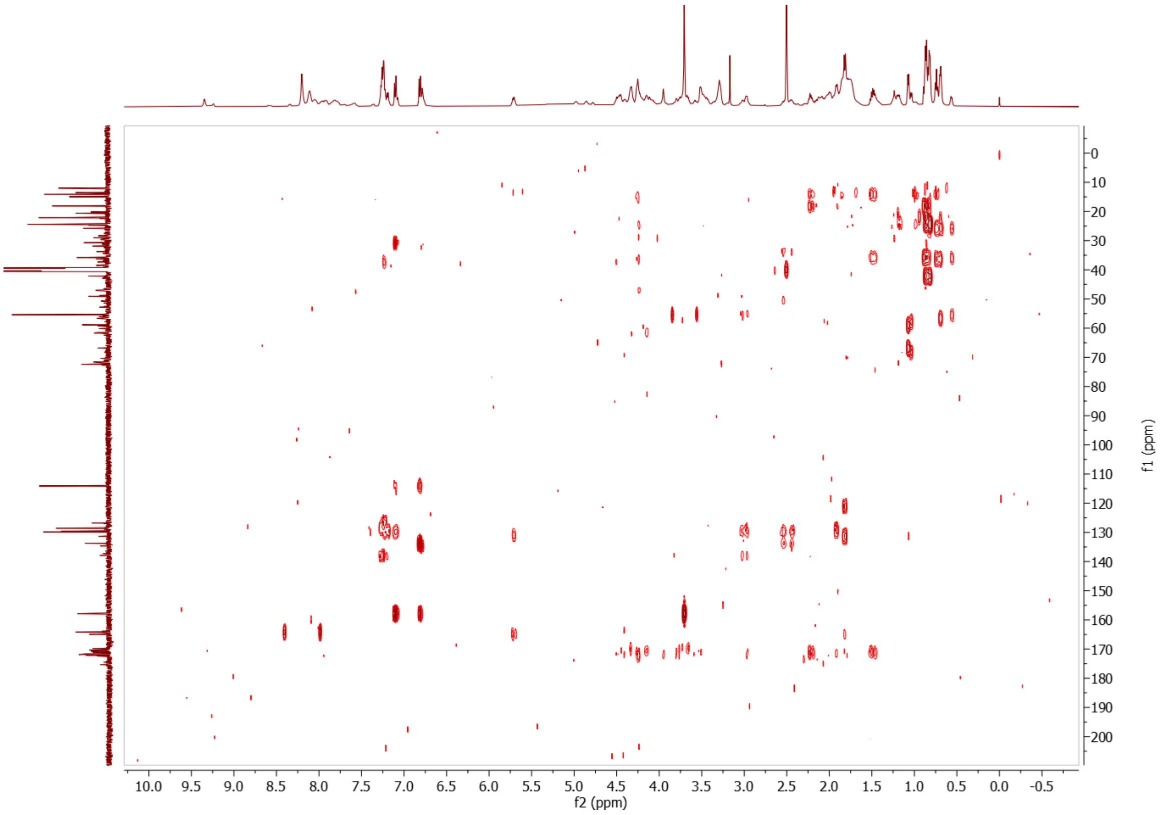

Figure S5. HMBC of floridanemamide A.

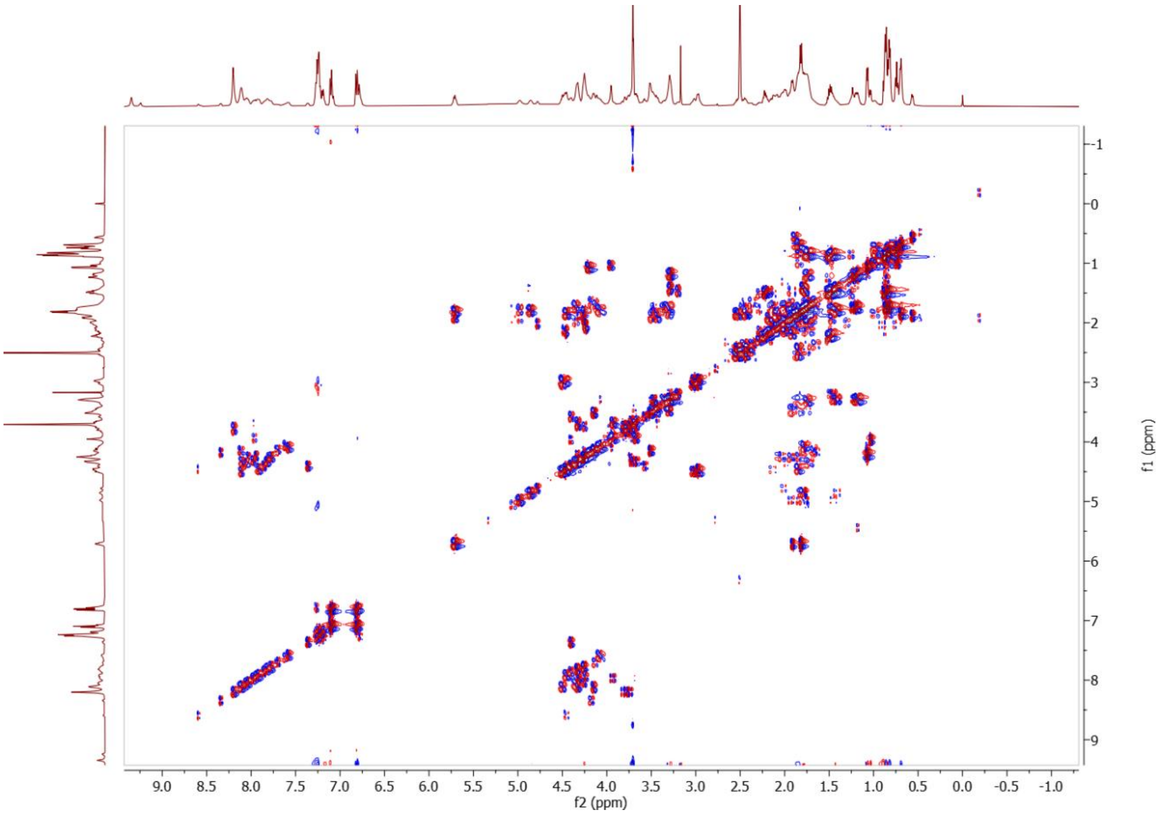

Figure S6. DQF-COSY of floridanemamide A.

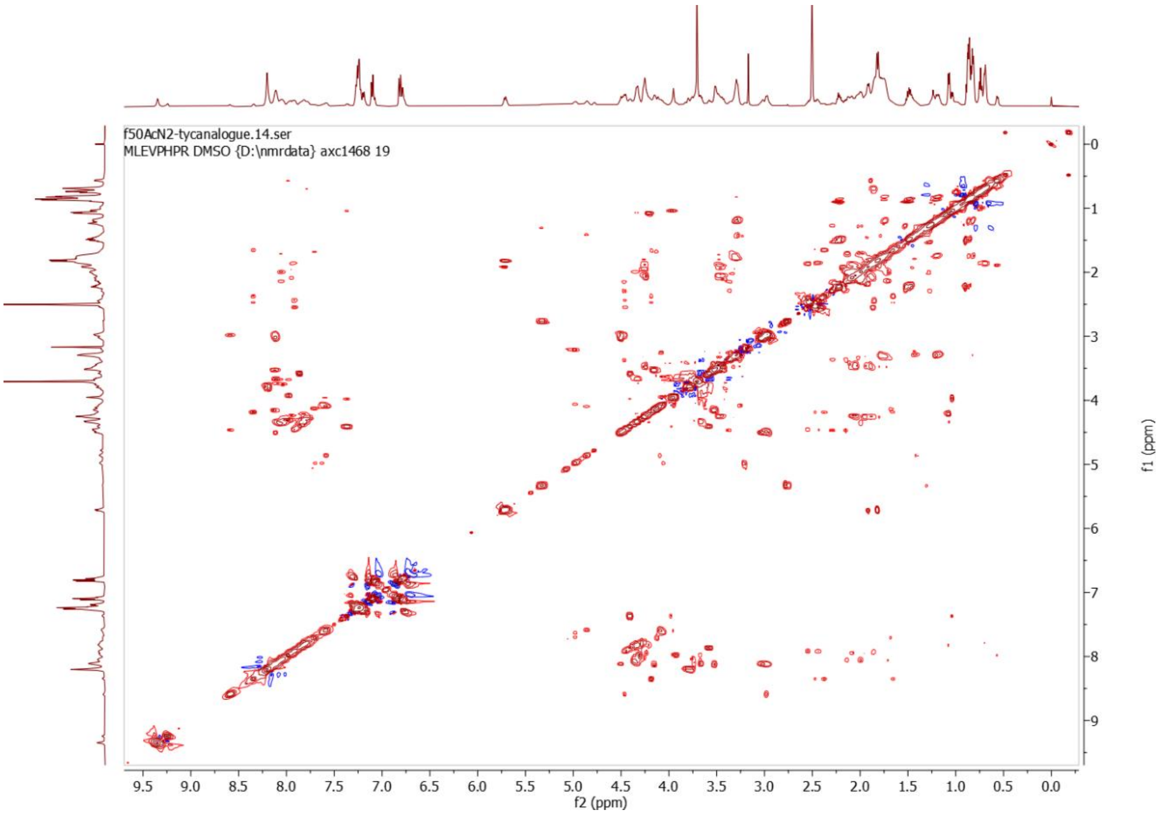

Figure S7. TOCSY of floridanemamide A.

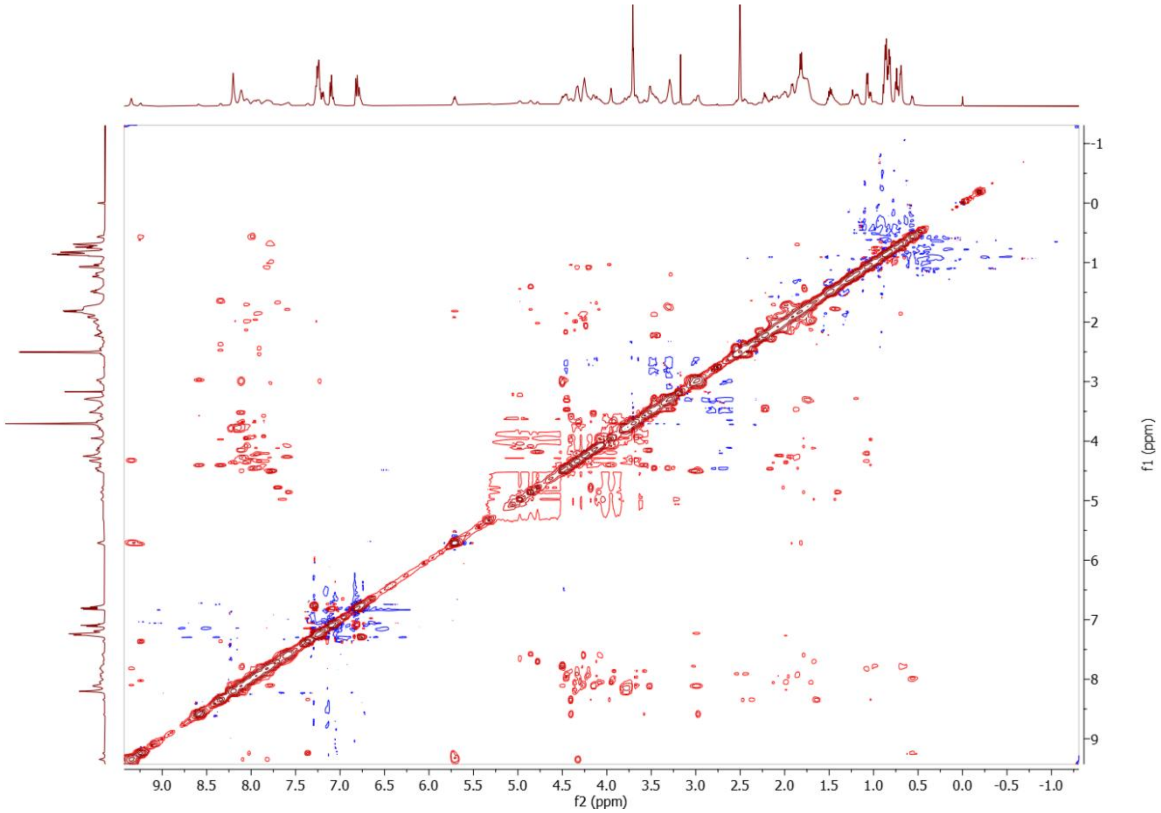

Figure S8. NOESY of floridanemamide A.

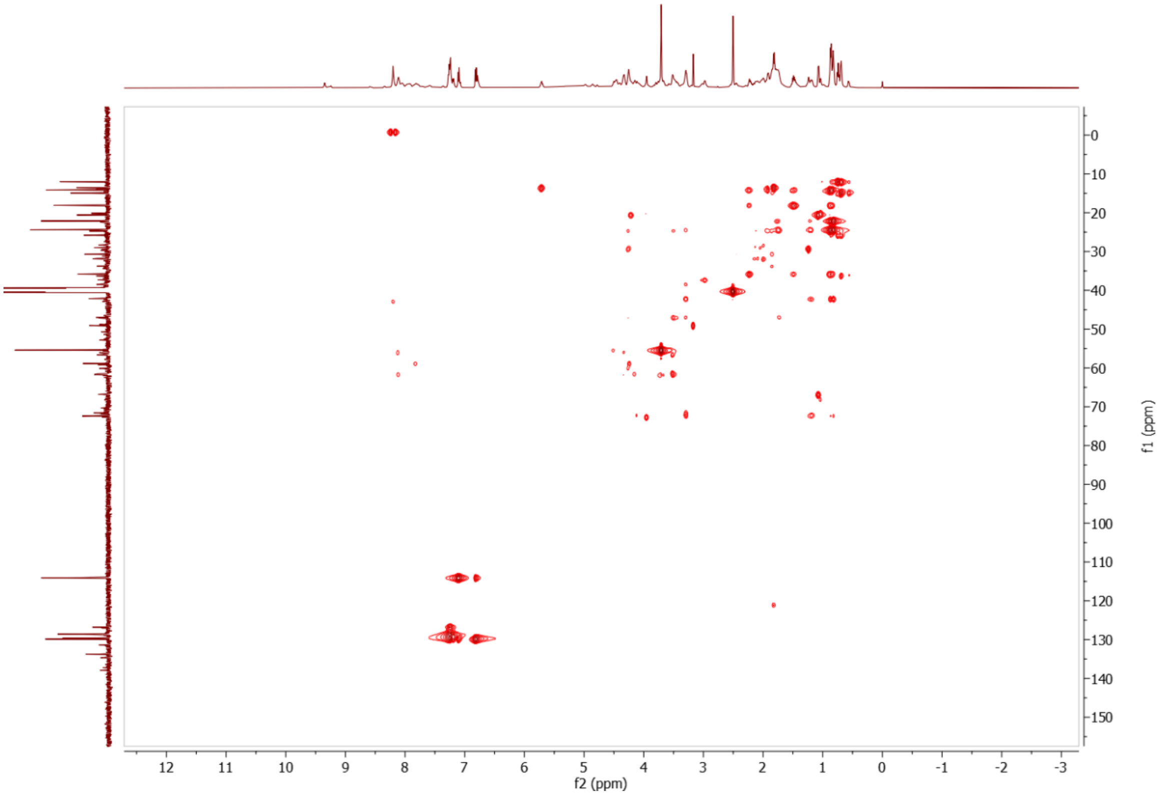

Figure S9. HSQC-TOCSY of floridanemamide A.

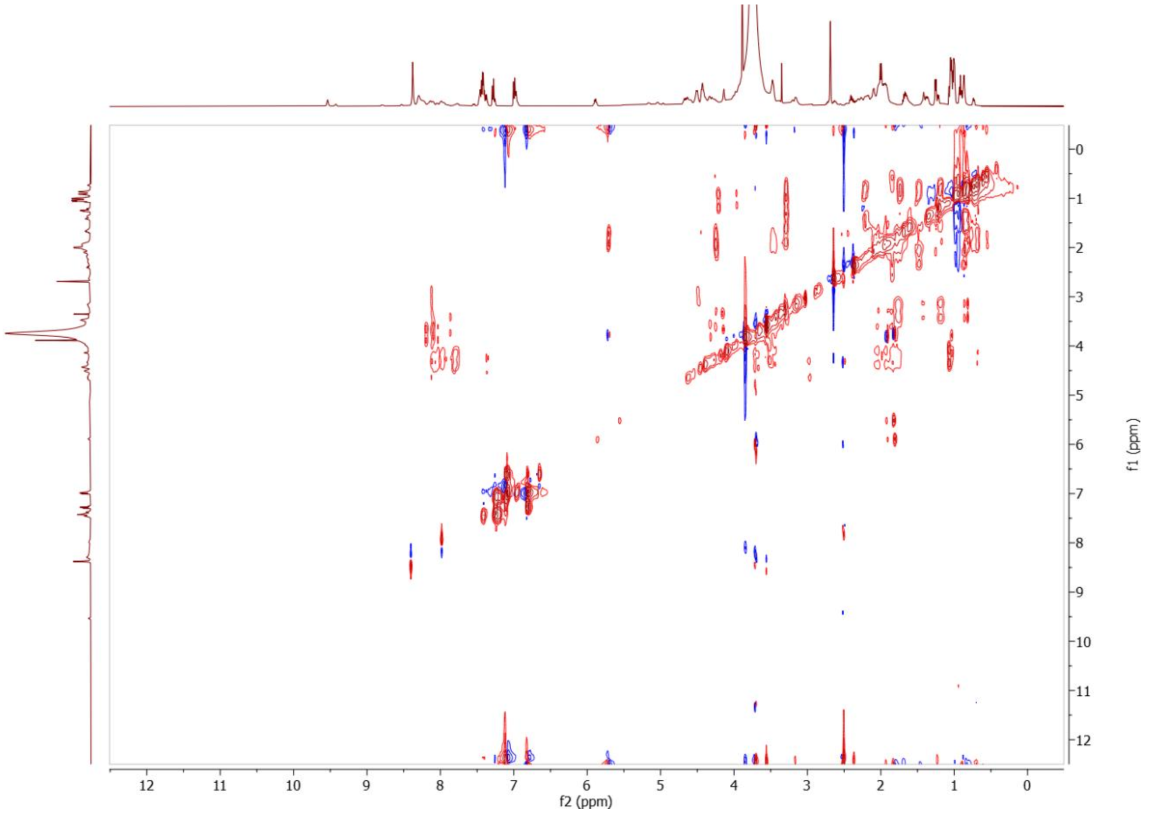

Figure S10. HETLOC of floridanemamide A.

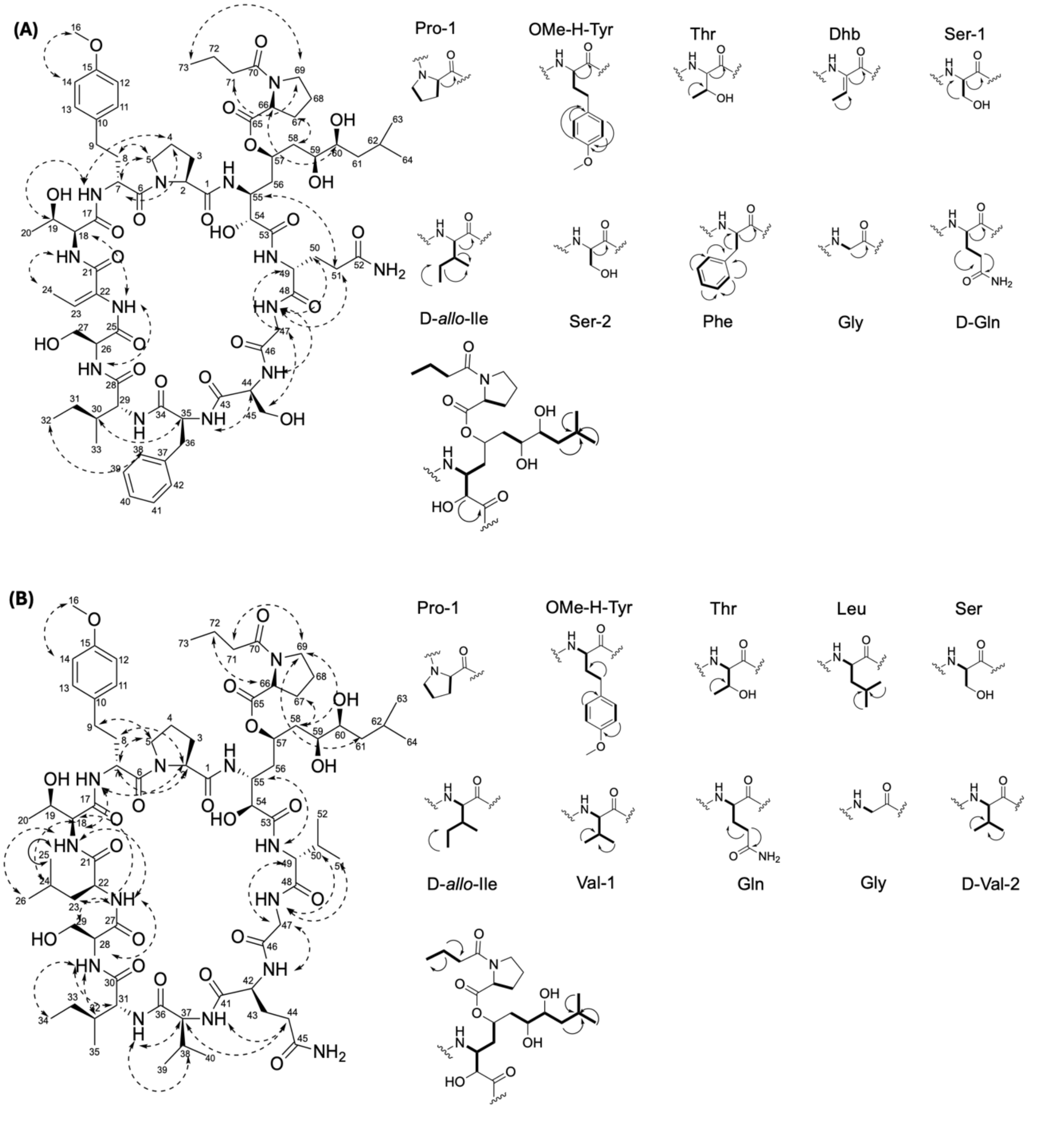

Figure S11. Select 2D NMR correlations for (A) floridanemamide A (**1**) and (B) floridanemamide C (**3**). TOCSY correlations shown with bold lines, HMBC correlations shown with arrows and NOE correlations shown with dashed arrows.

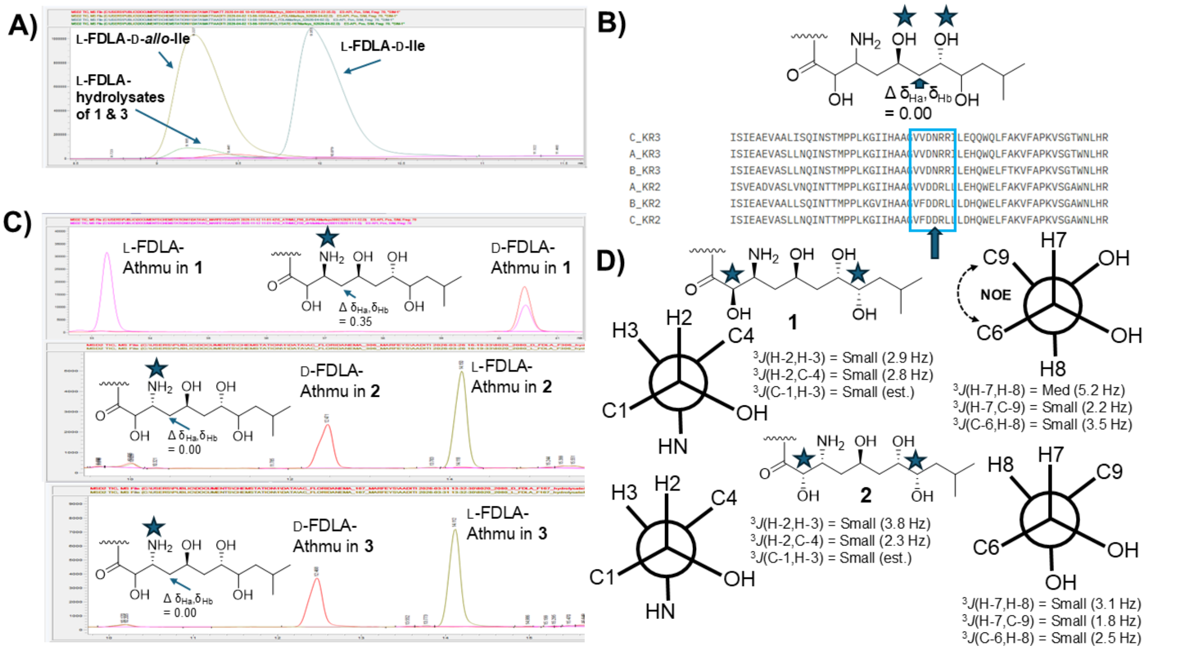

Figure S12. Relative and absolute configuration analysis of **1-3**. (A) Marfey's analysis of the hydrolysates of **1** and **3** reacted with L-FDLA and compared to L-FDLA reacted standards. (B) Sequence analysis of the KR domains predicted to reduce β-ketones in the *fma* pathway with the VDD sequence indicative of B-type KRs and the VDN sequence indicative of A-type KRs noted in the blue box. Additionally, the difference between the two protons attached to the intervening methylene unit is noted, which was 0.00 for **1-3** (blue arrow). (C) The determination of C-3 configuration of the Athmu unit by reacting the hydrolysates of **1-3** with L- and D- FDLA, respectively. Additionally, the difference between the two protons attached to the intervening methylene unit between C-3 and C-5 is noted for **1-3**, which was 0.35 for **1** and 0.00 for **2** and **3**. (D) *J*-coupling analysis of portions the Athmu unit to determine the relative configuration between C2 and C3 and C7 and C8 in **1** and **2**. The coupling constants for **3** exhibited medium (4.6 Hz), small (2.5 Hz), and small (est.) respective *J* values for H2-H3, H2-C4 and H3-C1 and small, small, small values for H7-H8, H7-C9, and H8-C6, respectively.

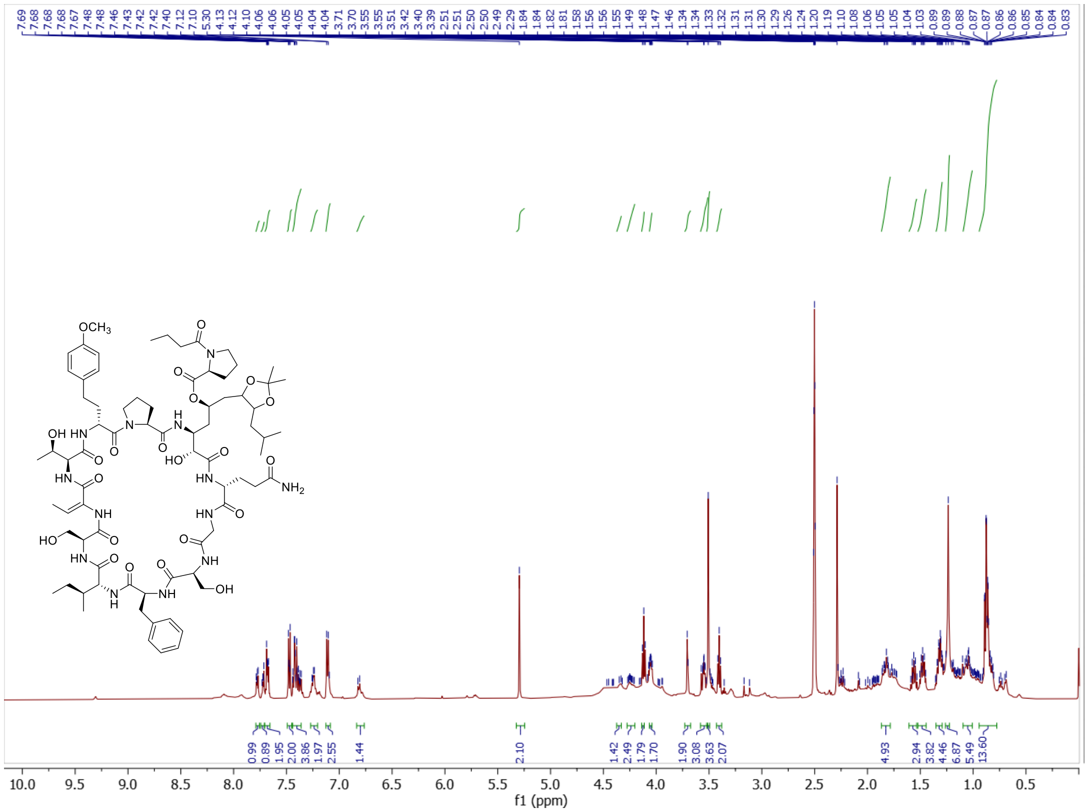

Figure S13. ^{1}H NMR of the floridanemamide A acetonide derivative (500 MHz, DMSO-d_6).

Floridanemamide/tychonamide partial gene cluster from cyanobacterial bloom sample

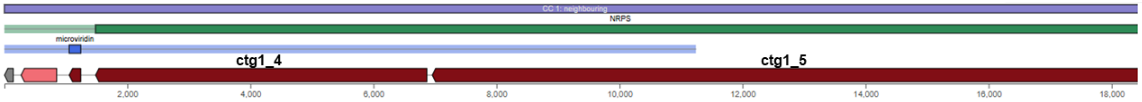

Protein modules and domains

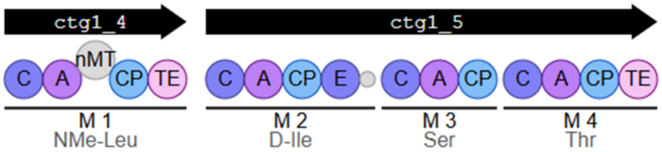

Closest NCBI BLASTP hit

	Cluster Composition<br>Click the ⊞ to see the cluster contents	Cluster Ancestor	Cluster Representative Sequence	Max Score	Total Score	Query Cover	E value	Per. Ident	Acc. Len	Accession
☐	⊞ 3 member(s), 3 organism(s)	cyanobacteria	amino acid adenylation domain-containing protein [Floridanema aerugi...	3694	3694	100%	0.0	100.00%	1795	WP_413268529.1

Figure S14. Partial antiSMASH (version 8) annotation of partial floridanemamide/tychonamide pathway in Lake Erie, Showse Park MAG. Protein modules and domains are shown below the open reading frames and the closest BLASTP hit for contig1_4 was *Floridanema aerugineum* with 100% amino acid identity with a 1795 accession length.

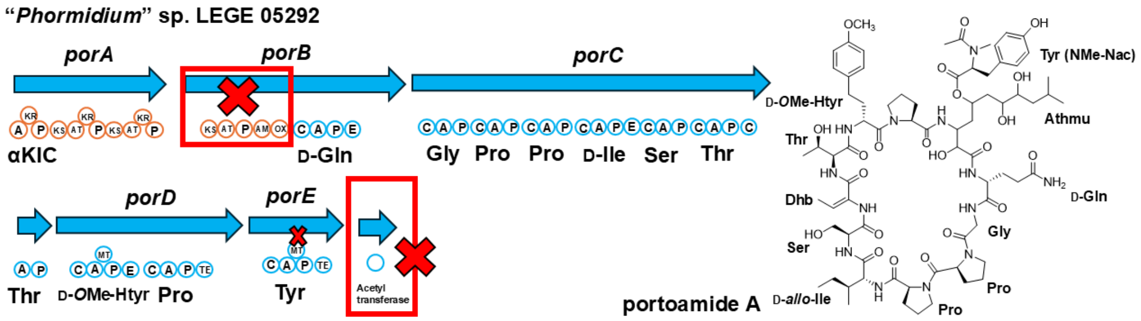

Figure S15. Putative portoamide (*por*) biosynthetic pathway with the portoamide A structure shown at right. Red boxes show portions of pathway that were not annotated but are predicted for biosynthesis. For abbreviations, see Figure 1 legend.

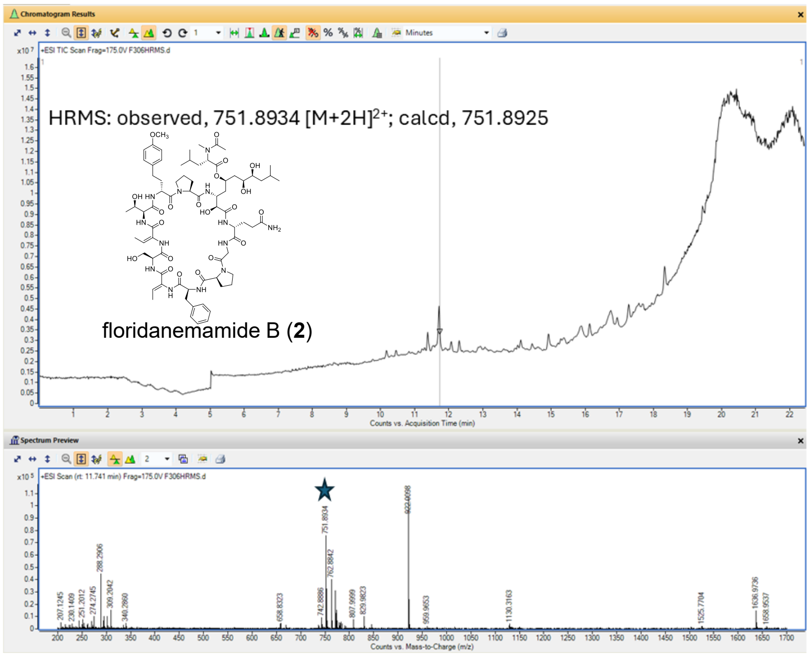

Figure S16. HRESIMS measurement of floridanemamide B (**2**) *m/z* 751.8934 $[M+2H]^{2+}$.

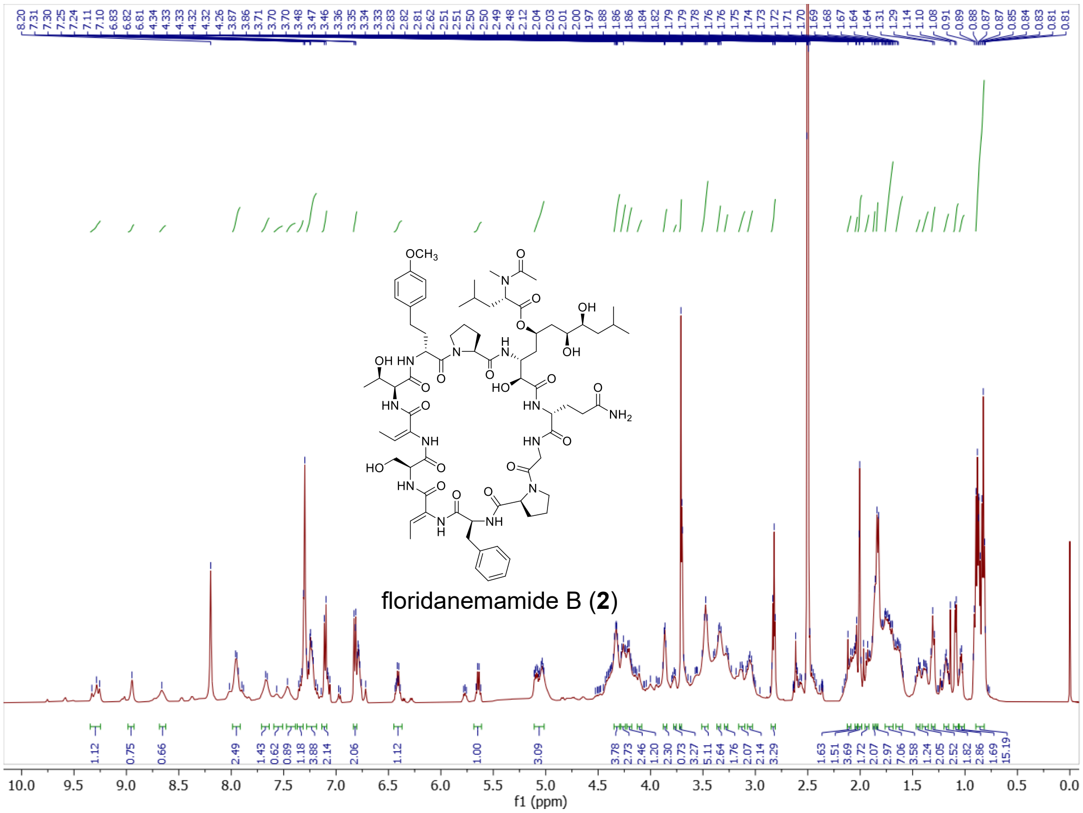

Figure S17. ^{1}H NMR of floridanemamide B (**2**) (500 MHz, DMSO-d_6).

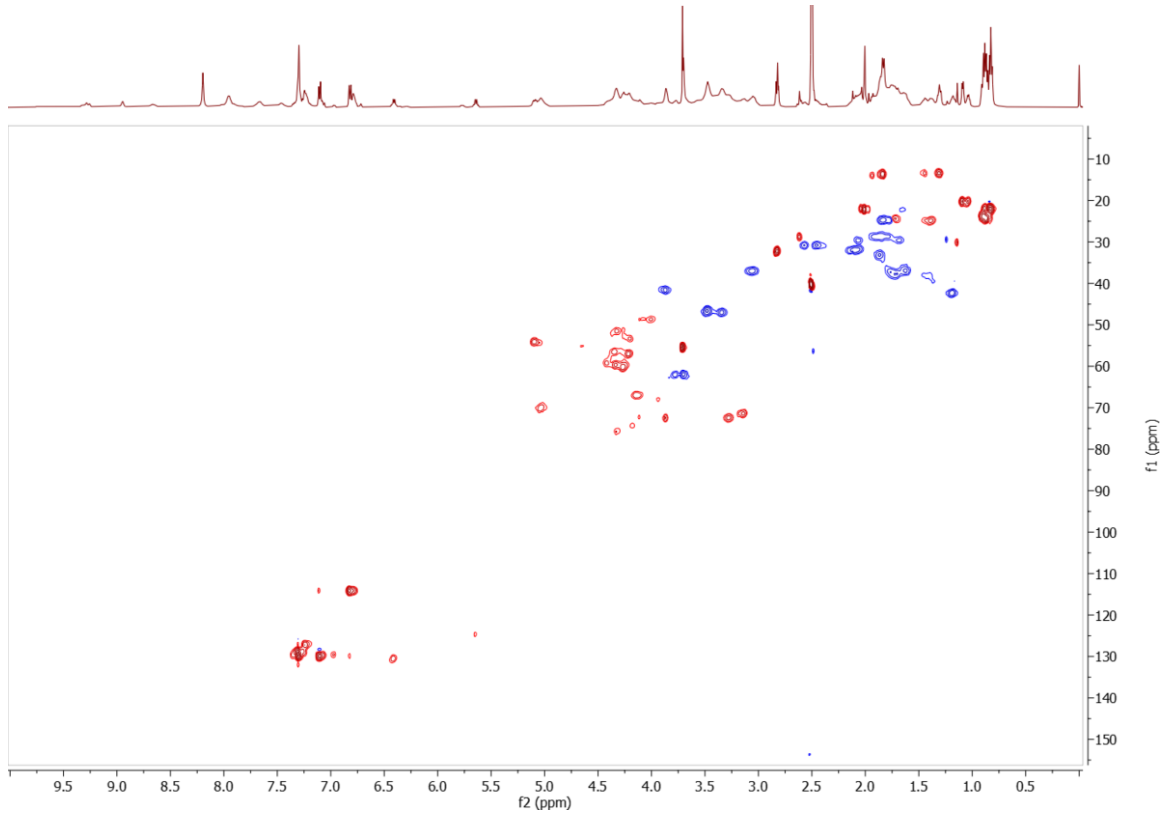

Figure S18. Multiplicity-edited HSQC of floridanemamide B.

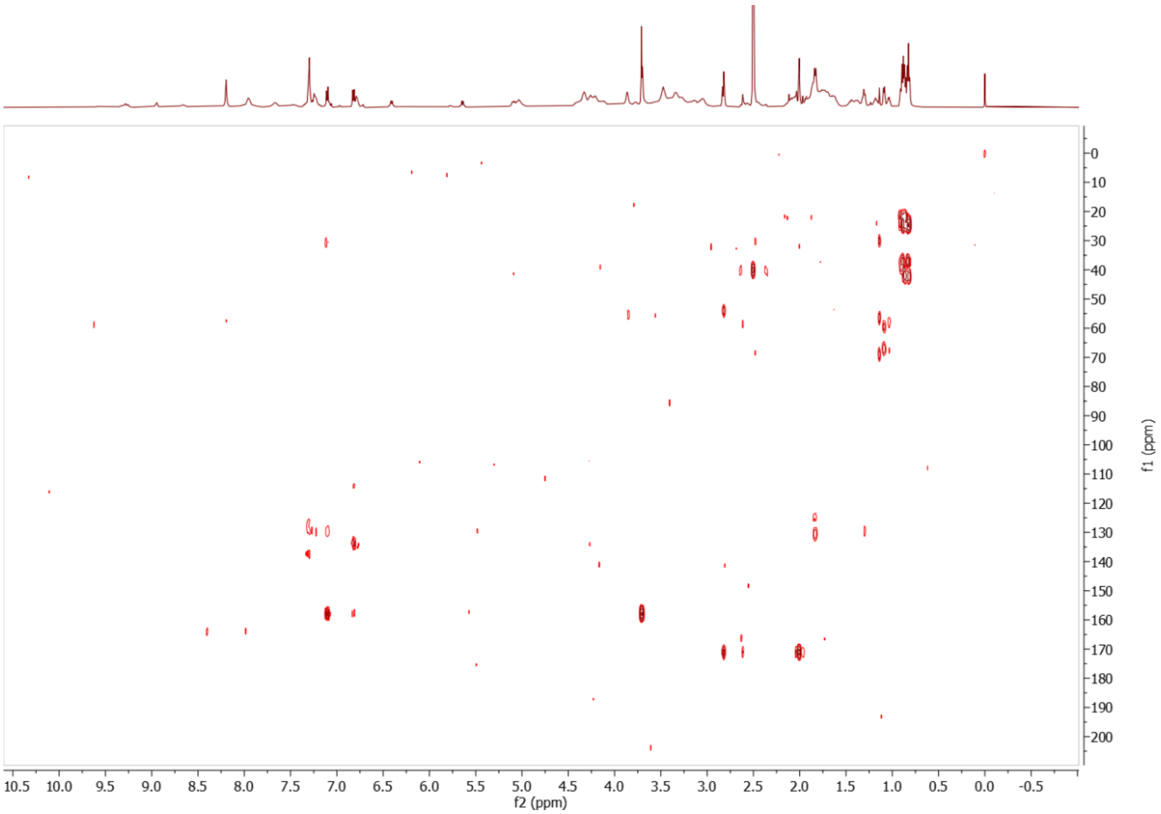

Figure S19. HMBC of floridanemamide B.

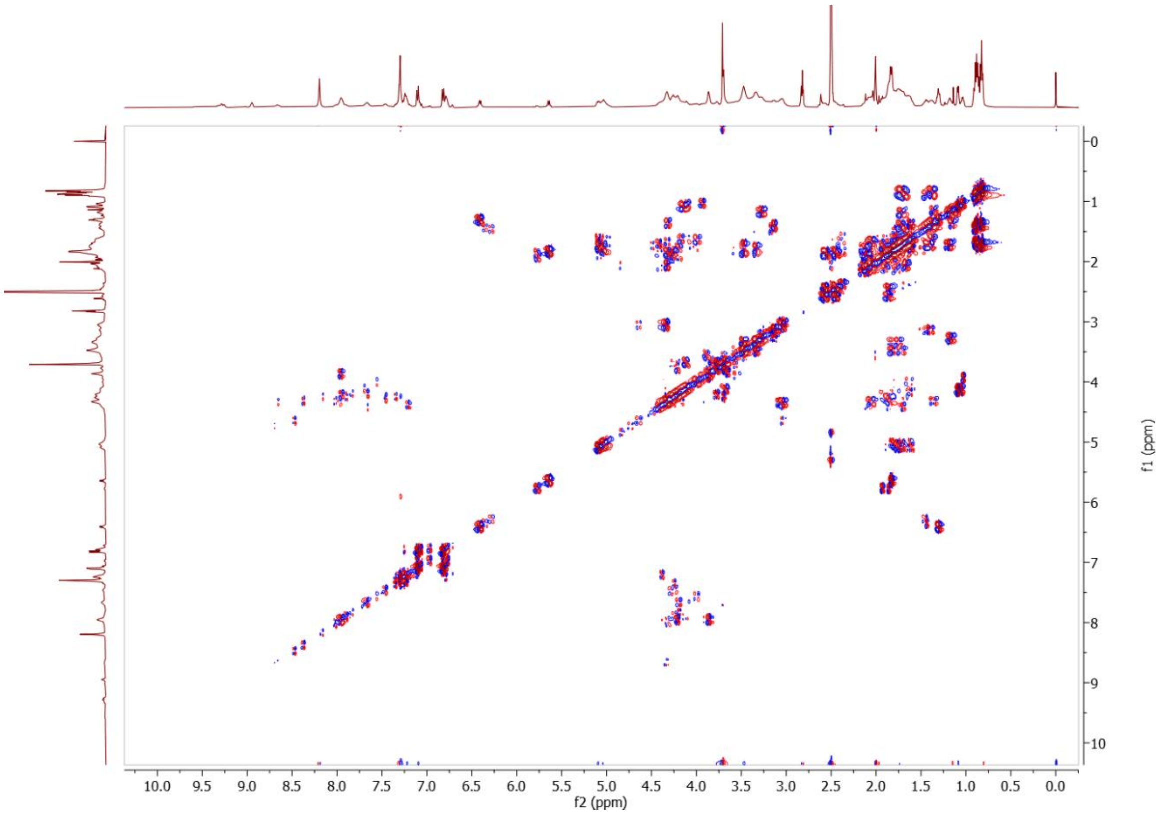

Figure S20. DQF-COSY of floridanemamide B.

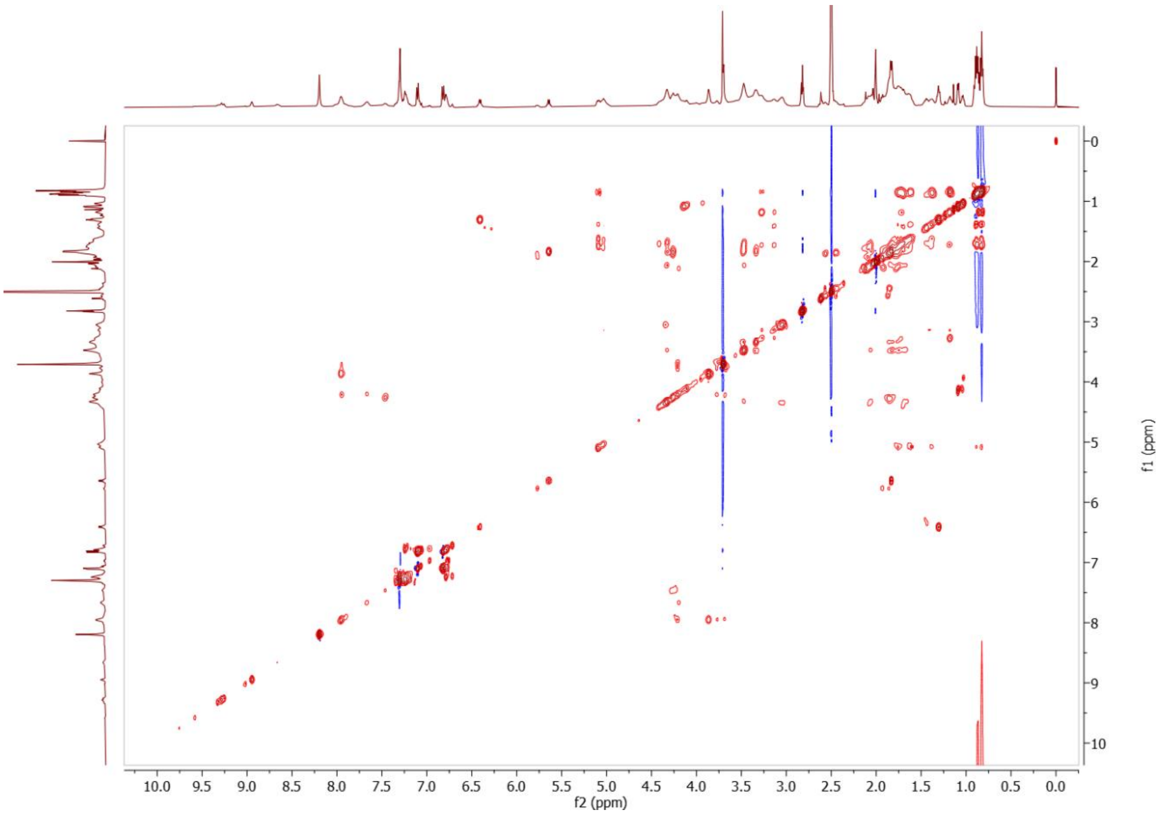

Figure S21. TOCSY of floridanemamide B.

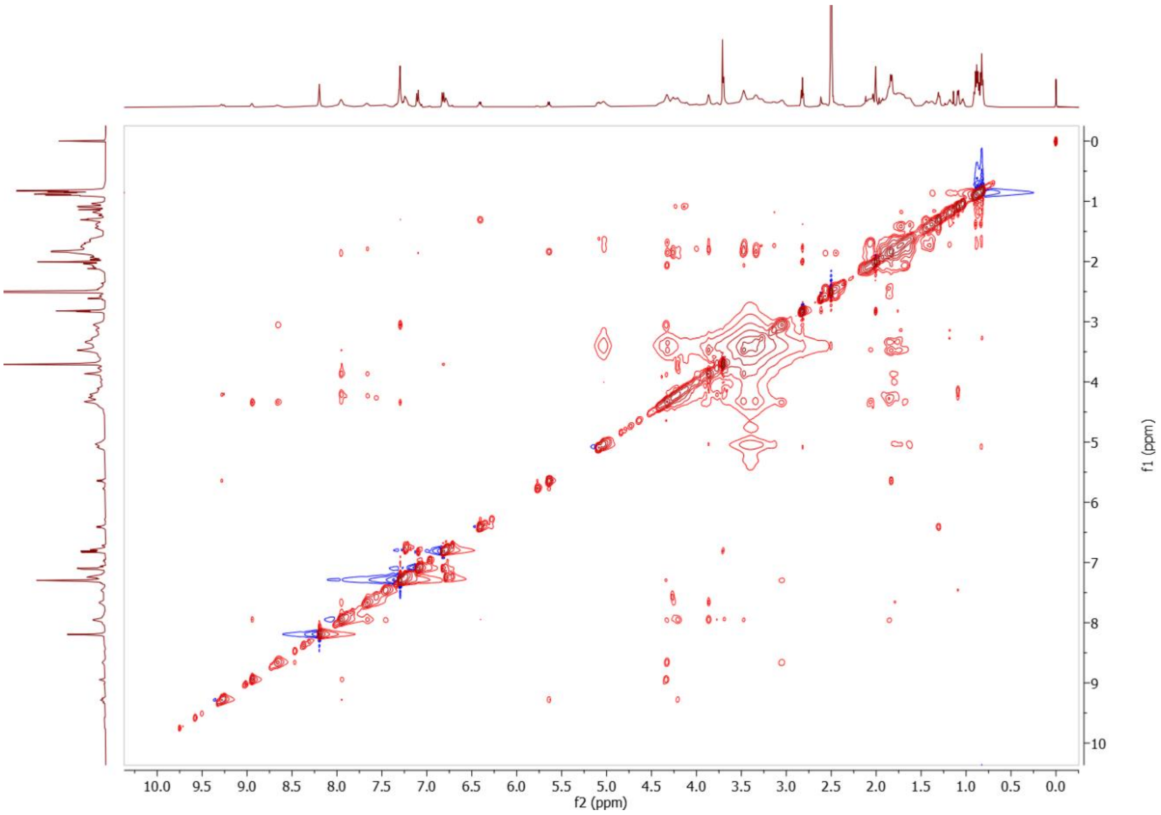

Figure S22. NOESY of floridanemamide B.

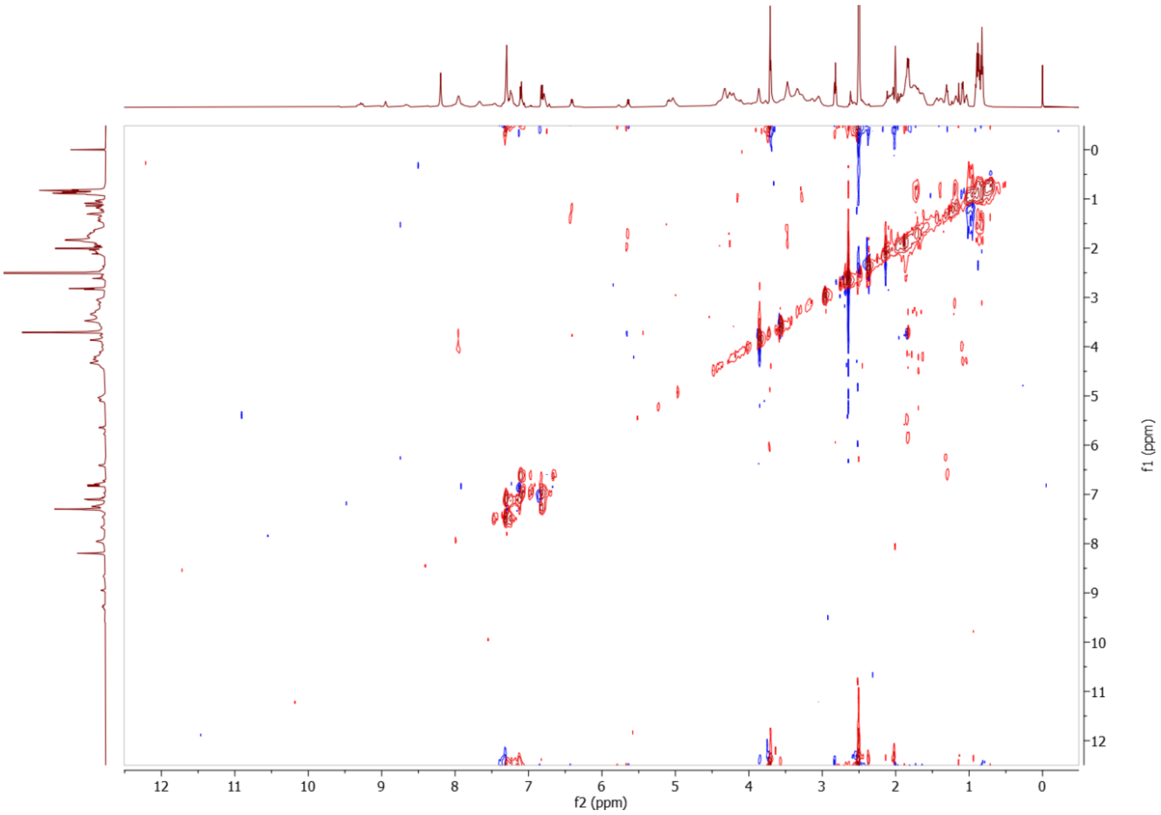

Figure S23. HETLOC of floridanemamide B.

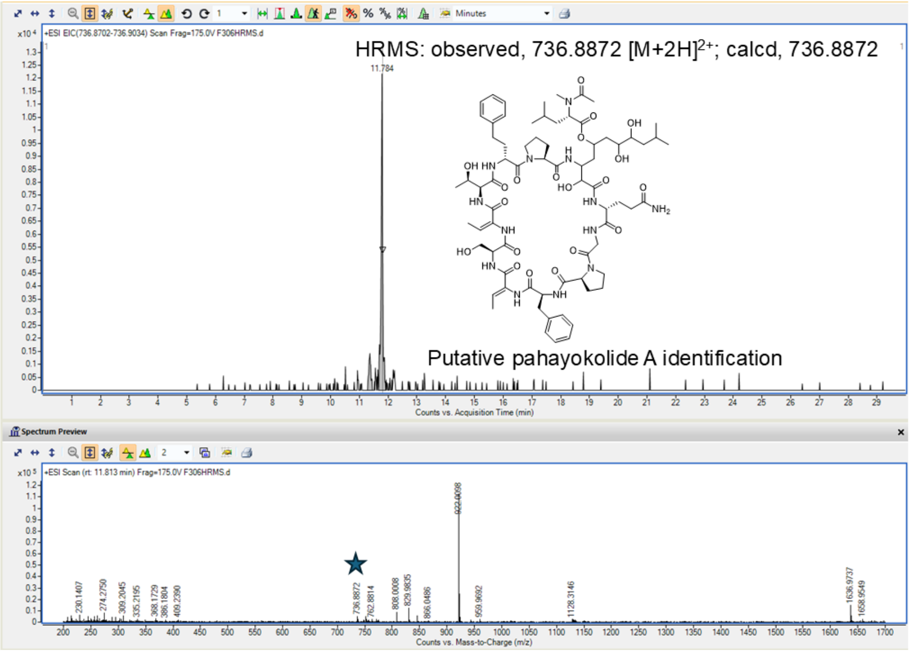

Figure S24. HRESIMS measurement of putative pahayokolide A *m/z* 736.8872 $[M+2H]^{2+}$.

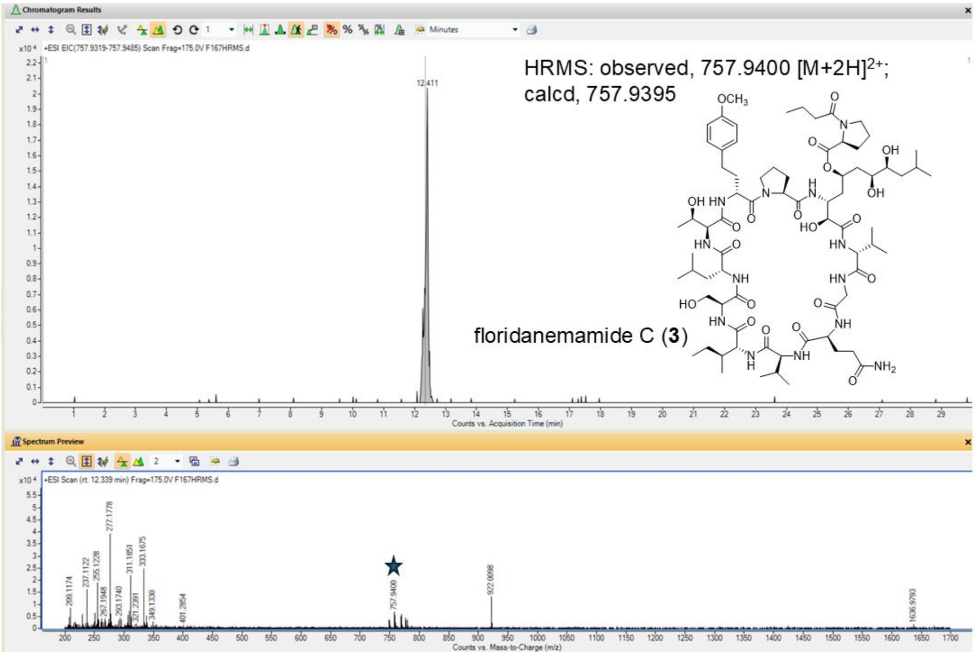

Figure S25. HRESIMS measurement of floridenemamide C (**3**) *m/z* 757.9400 $[M+2H]^{2+}$.

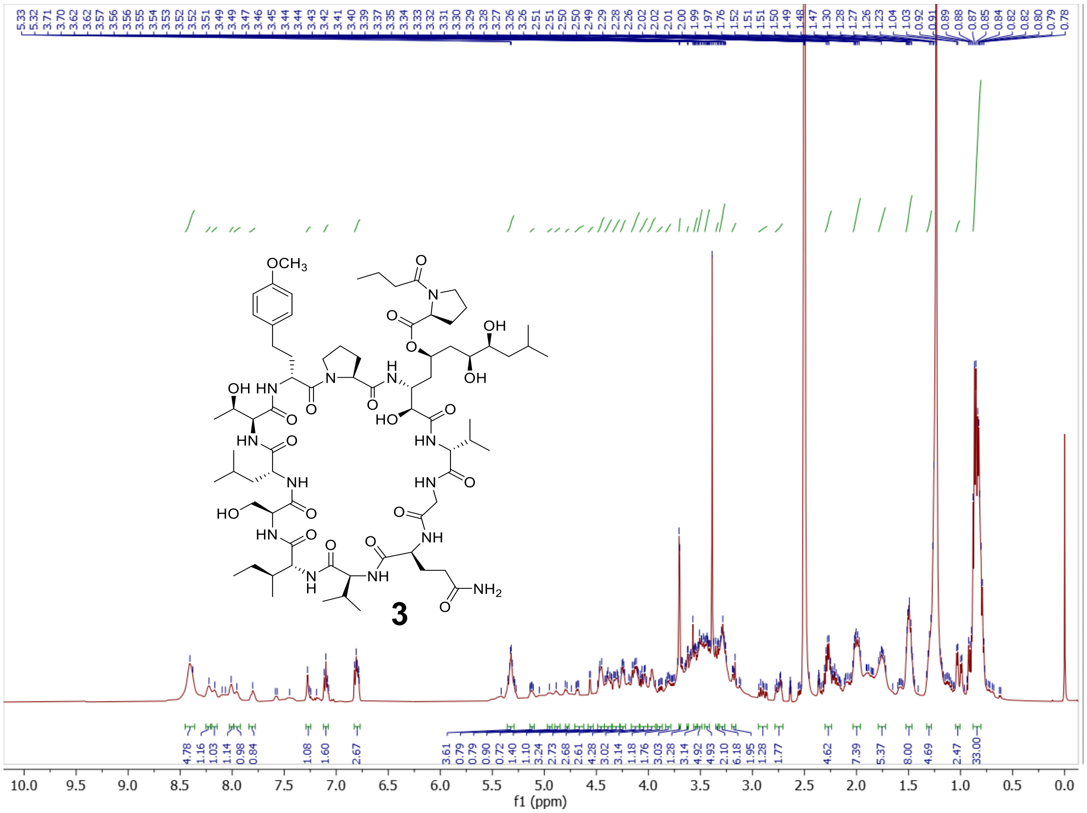

Figure S26. ^{1}H NMR of floridanemamide C (**3**) (500 MHz, DMSO-d_6).

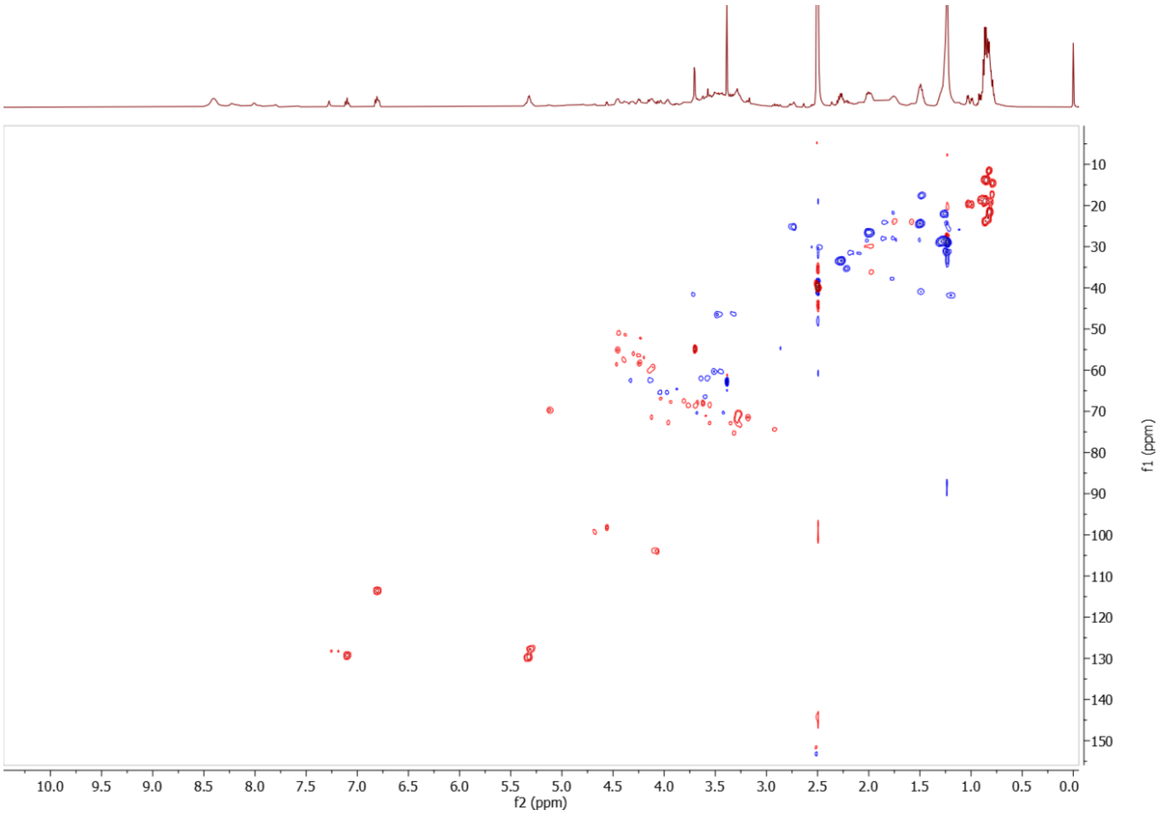

Figure S27. Multiplicity-edited HSQC of floridanemamide C.

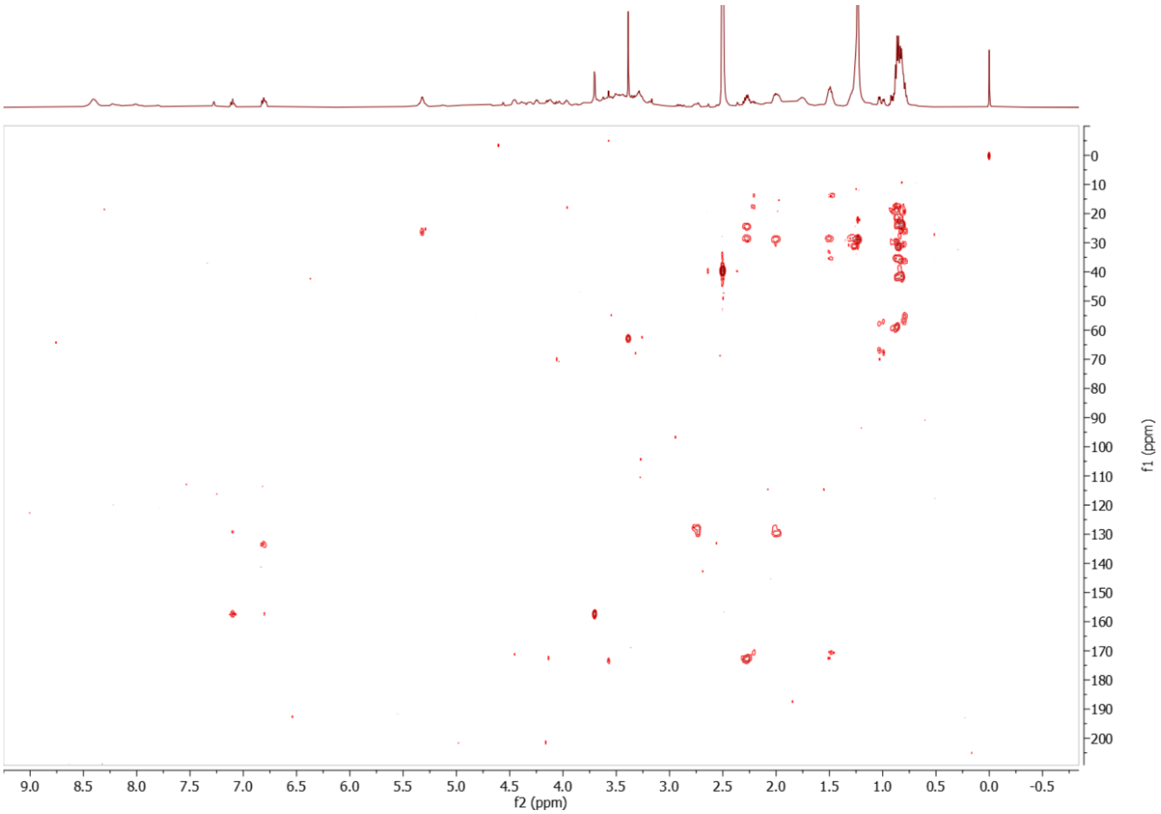

Figure S28. HMBC of floridanemamide C.

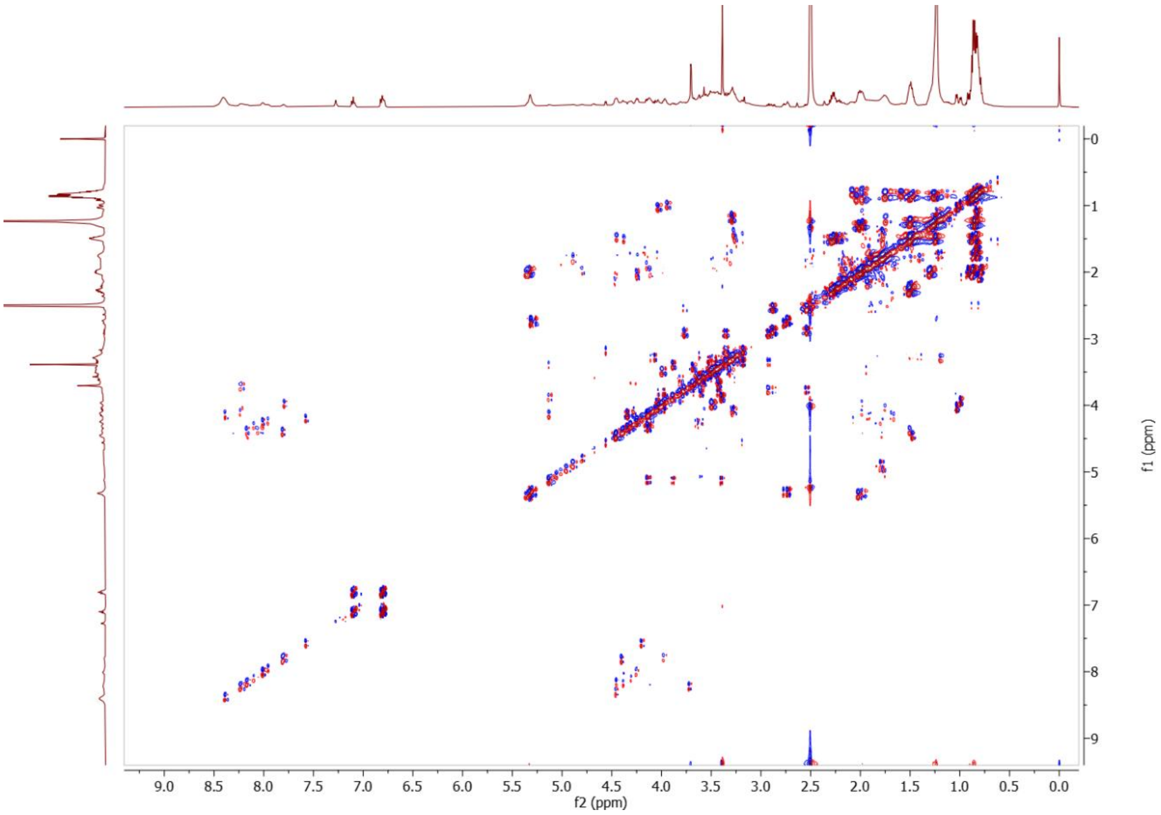

Figure S29. DQF-COSY of floridanemamide C.

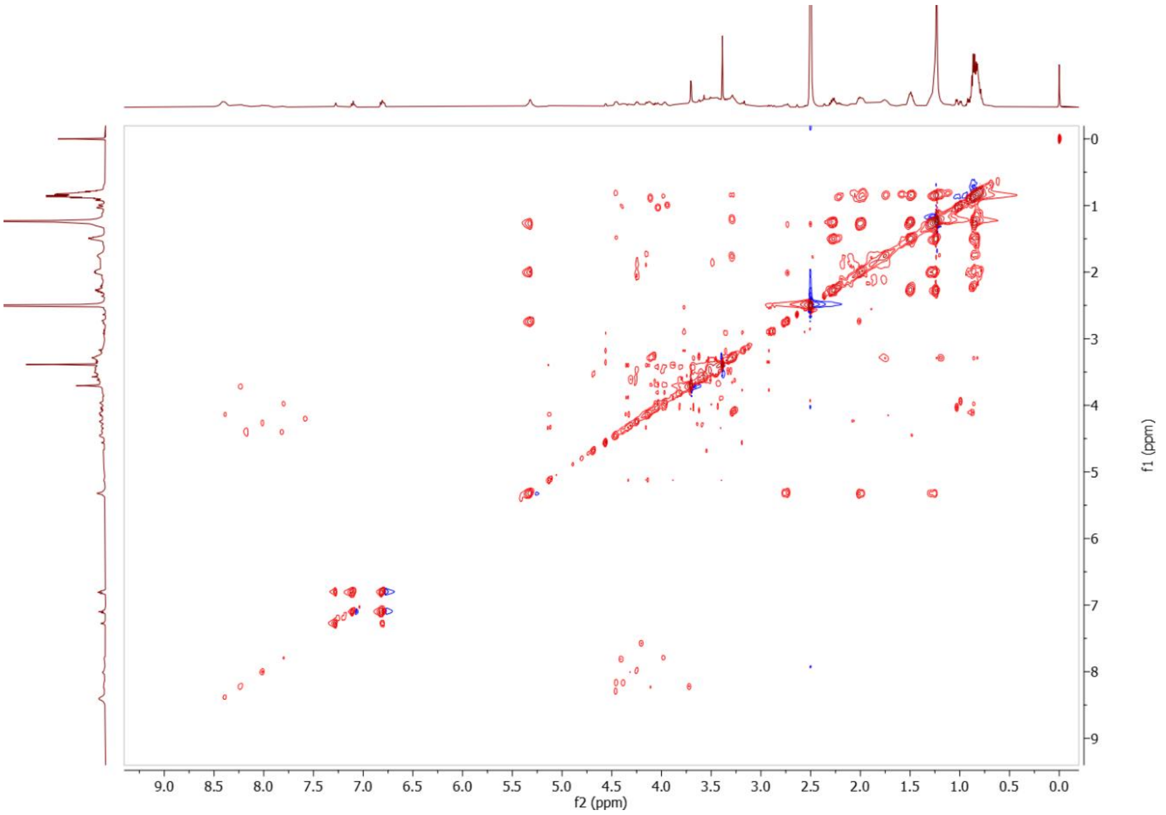

Figure S30. TOCSY of floridanemamide C.

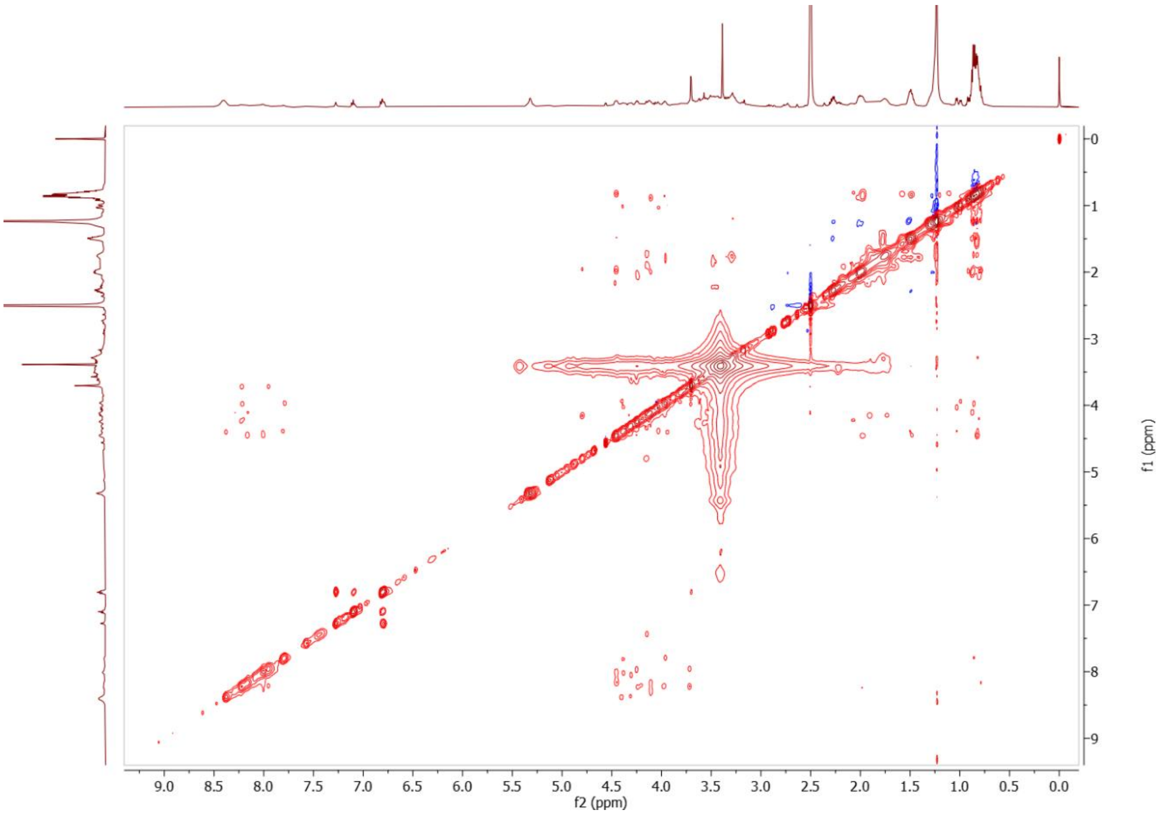

Figure S31. NOESY of floridanemamide C.

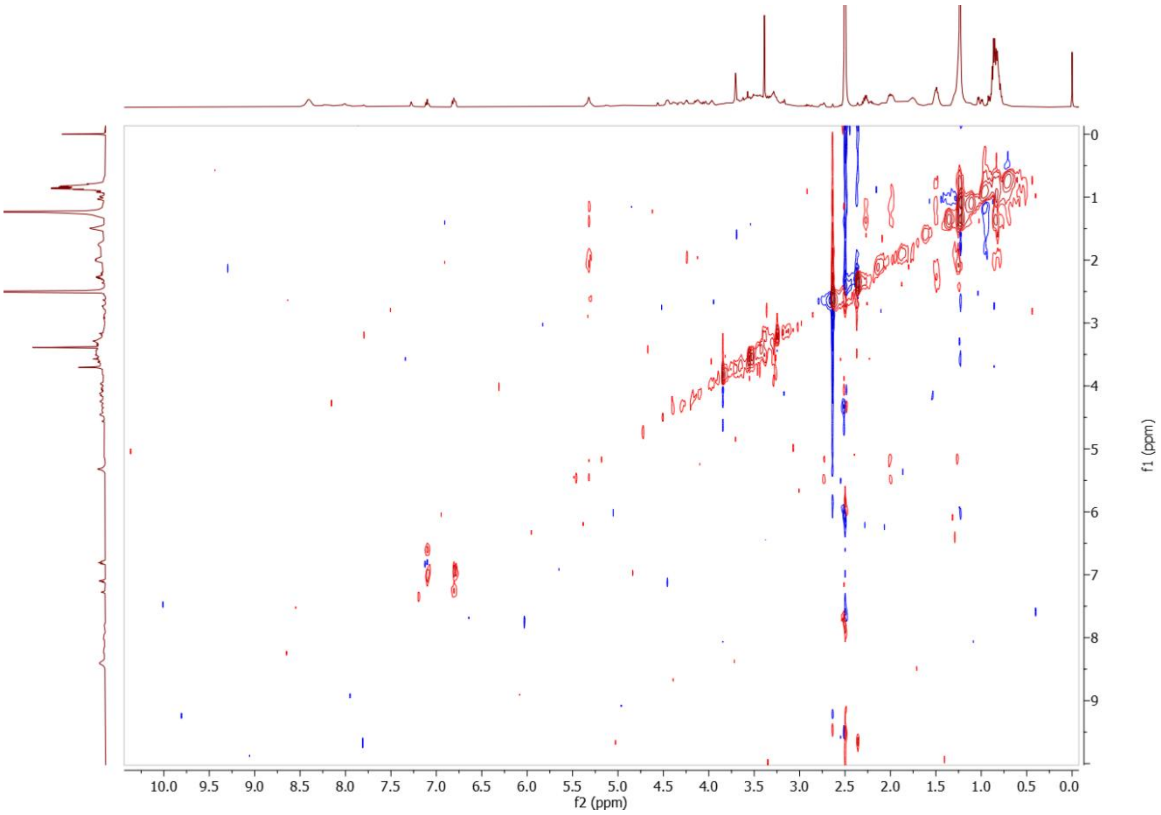

Figure S32. HETLOC of floridanemamide C.
